# Integrating highly quantitative proteomics and genome-scale metabolic modeling to study pH adaptation in the human pathogen *Enterococcus faecalis*

**DOI:** 10.1038/npjsba.2016.17

**Published:** 2016-09-08

**Authors:** Ruth Großeholz, Ching-Chiek Koh, Nadine Veith, Tomas Fiedler, Madlen Strauss, Brett Olivier, Ben C Collins, Olga T Schubert, Frank Bergmann, Bernd Kreikemeyer, Ruedi Aebersold, Ursula Kummer

**Affiliations:** 1 BioQuant, Centre for Organismal Studies (COS), Heidelberg University, Heidelberg, Germany; 2 Department of Biology, Institute of Molecular Systems Biology, ETH Zurich, Zurich, Switzerland; 3 Institute of Medical Microbiology, Virology and Hygiene, Rostock University Medical Centre, Rostock, Germany; 4 Amsterdam Institute for Molecules, Medicines and Systems, VU University Amsterdam, Amsterdam, Netherlands; 5 Faculty of Science, University of Zurich, Zurich, Switzerland

## Abstract

Genome-scale metabolic models represent the entirety of metabolic reactions of an organism based on the annotation of the respective genome. These models commonly allow all reactions to proceed concurrently, disregarding the fact that at no point all proteins will be present in a cell. The metabolic reaction space can be constrained to a more physiological state using experimentally obtained information on enzyme abundances. However, high-quality, genome-wide protein measurements have been challenging and typically transcript abundances have been used as a surrogate for protein measurements. With recent developments in mass spectrometry-based proteomics, exemplified by SWATH-MS, the acquisition of highly quantitative proteome-wide data at reasonable throughput has come within reach. Here we present methodology to integrate such proteome-wide data into genome-scale models. We applied this methodology to study cellular changes in *Enterococcus faecalis* during adaptation to low pH. Our results indicate reduced proton production in the central metabolism and decreased membrane permeability for protons due to different membrane composition. We conclude that proteomic data constrain genome-scale models to a physiological state and, in return, genome-scale models are useful tools to contextualize proteomic data.

## Introduction

Genome-scale models link genomic information and experimentally observable biological phenotypes by allowing *in silico* experiments on a whole-cell scale.^1^ For instance, a model of *Lactococcus lactis* MG1363 provided insights into mechanisms of flavor formation, which is of high relevance and importance to the dairy industry.^2^ Similarly, a model representing the human pathogen *Listeria monocytogenes* revealed new aspects of the connection between virulence and metabolism.^3^

Typically, genome-scale models represent a rather static view of the organism’s metabolism: protein products of all genes encoded in the genome are considered to be active simultaneously. In reality, limitations in space, physical capacities or energetic constraints usually restrain gene expression as a function of environment, genetics, time and other factors.

In genome-scale models such limitations have typically been identified from by transcriptomic data (where messenger RNA (mRNA) levels serve as proxy for protein levels)^4–8^ and new methods were developed to integrate this information.^5–10^ Integration of gene expression leads either to binary decisions,^9,10^ often involving reactivation of certain reactions (e.g., MADE^9^), or heuristically determined changes in the flux bounds, e.g., E-Flux.^8^ However, transcript levels often represent the levels of their corresponding proteins poorly, particularly if perturbation induced changes are considered. mRNA levels explain only 29 to 55% of the protein levels in prokaryotes^11^ and 27% in human cells.^12,13^ Because this low correlation does not allow us to predict protein concentrations accurately, we expect that proteomic data constrains genome-scale models more effectively than transcript data. Constraining models generally leads to a limitation of the solution space, which can be quite large, and therefore constraints can help to increase the robustness of the results. Until recently it has been challenging to generate suitable proteomic data sets whereby a large fraction of the proteome is reproducibly detected and accurately quantified. A newly developed mass spectrometric (MS) technique, SWATH-MS can quantify a substantial fraction of the proteome with high reproducibility and at high throughput.^14,15^ This technique has been successfully applied to study dynamics during dormancy and resuscitation in *Mycobacterium tuberculosis* facilitating identification of potential drug targets.^16^

Although protein data have served as a basis for cell-type-specific genome-scale reconstruction before,^17,18^ only few studies integrated state-specific proteomic data into a genome-scale model.^19,20^

We describe a method to integrate whole-cell proteomic data into genome-scale models, facilitating both mapping of detected proteins and concentration changes thereof to constrain the solution space. To validate our method we obtained quantitative proteomic data for the pathogen *Enterococcus faecalis* V583,^21,22^ sampled during a pH shift experiment.

*E. faecalis* quickly adapts to changing environmental conditions and resides in diverse native environments.^21,22^ The intrinsic versatility of *E. faecalis* enables it to survive extremely harsh conditions such as high temperature, salinity and alkaline pH.^22,23^ Specifically, in response to a decreasing environmental pH *E. faecalis* changes its fermentation pattern^24^ and increases the activity of the H^+^ translocating V-type ATPase.^25,26^

**Figure 1:**
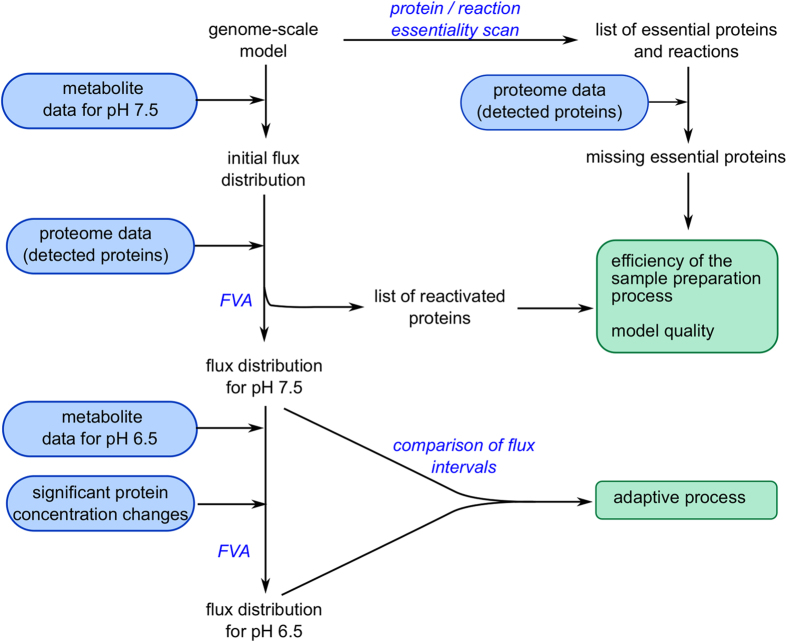
Schematic overview of our data integration approach. Experimental parts are indicated in blue boxes, the main outputs are shown in green boxes, computational methods are presented in blue script. FVA, flux variability analysis.

We investigated the adaptive processes of *E. faecalis* V583 to a decreasing environmental pH by combining quantitative whole-cell proteomic data with genome-scale modeling. Specifically, we integrated the proteomic data into the validated genome-scale model of this organism^27^ inactivating undetected proteins (29% of the proteins annotated in the model) and applying significant protein concentration changes in the form of flux constraints. We further measured metabolite consumption and production rates throughout the experiment and used these to compute flux distributions to compare the different metabolic states (Figure 1). Furthermore, using flux variability analysis (FVA) we were able to observe and contextualize adaptive processes to a more acidic environmental pH such as a change in fermentation pattern and an increase in ATPase activity. Last, the proteomic data allowed us to infer structural adaptations during the pH shift and suggest regulatory mechanisms on the level of enzyme activity. In conclusion, we show that genome-scale models are useful tools to contextualize proteomic data and that, in return, genome-scale models benefit from the constraints imposed by the proteomic data to give a more physiologically relevant solution.

## Results

### Effect of pH on biomass and metabolism

**Figure 2:**
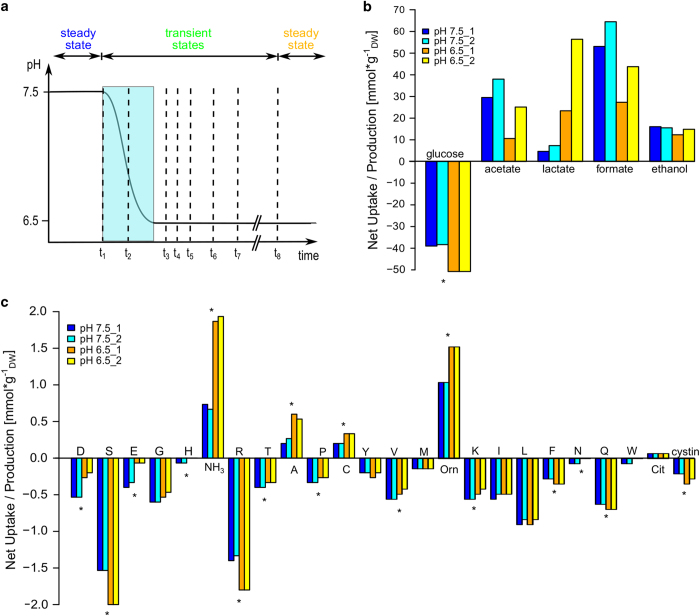
Experimental setup and metabolite measurements. Uptake and production were calculated as $\left(\left[C_{\mathrm{chemostat}}\right]-\left[C_{\mathrm{medium}}\right]\right)/X$, where *C* represents the metabolite concentration (mmol l^−1^) and *X* the dry weight of the cells (g_DW_ l^−1^). Fluxes are calculated by multiplication with the dilution rate (*d*=0.15 h^−1^). (**a**) Experimental setup. The pH was shifted from 7.5 to 6.5 over the course of an hour. Samples were taken at the steady state at pH 7.5 (*t*_1_), during the pH shift (*t*_2_) at pH 7.0 after 30 min, as well as 80 min, 100 min, 120 min, 180 min, 240 min, and 21 h (t_3–8_) after the initializing the pH shift. (**b**) Carbohydrate measurements. The measured uptake and production (given in $\mathrm{mmol}\;g_{\mathrm{DW}}^{-1}$) are determined in continuous fermentation of the chemically defined medium. (**c**) Amino-acid measurements. The measured uptake and production (given in $\mathrm{mmol}\;g_{\mathrm{DW}}^{-1}$) are determined in continuous fermentation of the chemically defined medium. Cit, citruline; Orn, ornithine. Chemostat 1 and 2 represent biological replicates. Metabolites with significant changes between the steady states are indicated by *P<0.05. The flux boundaries derived from the metabolite measurements are listed in Supplementary Table S5 (pH 7.5) and Supplementary Table S6 (pH 6.5).

To examine the effects of a more acidic environment on *E. faecalis* V583, we designed a pH shift experiment in continuous culture under glucose-limited conditions, where we sampled both proteins and metabolites of two biological replicate cultures (Figure 2a). We observed a decrease in biomass from 1.98 to 1.51 g_DW_ l^−1^ upon the pH shift, which was associated with an increased glucose uptake rate (Figure 2b, Supplementary Tables S5,S6). As lactic acid bacteria in anaerobic conditions rely on glycolysis for energy generation, higher glucose uptake rates indicate higher ATP production rates, suggesting increased energy demand at pH 6.5. The change in biomass was accompanied by a change in fermentation pattern from mixed acid to mainly homolactic fermentation, confirming the previously observed pH dependence of the metabolic switch between homolactic and mixed acid fermentation in *E. faecalis*.^24^

At the same time, we observed increased uptake rates for L-arginine and L-serine during the pH shift (Figure 2c). Both amino acids are used for energy generation and the role of L-arginine in the adaptive response to decreasing extracellular pH values has already been discussed for other lactic acid bacteria.^28^

### Establishing a link between genome-scale model and environmental pH

**Figure 3:**
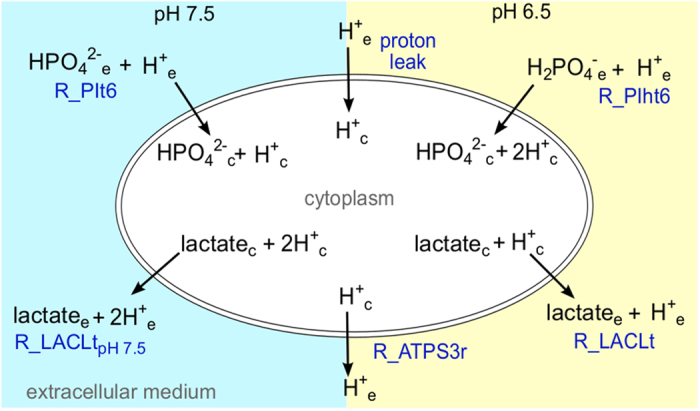
Proton-dependent reactions altered by the pH shift experiment as implemented in the genome-scale model. The state at pH 7.5 is highlighted in blue, pH 6.5 in yellow. R_PIt6/R_PIht6, phosphate transport via H^+^ symport at pH 7.5 and 6.5, respectively; R_LACLt_pH7.5_/R_LACLt, L-lactate transport via H^+^ symport at pH 7.5 and 6.5, respectively; R_ATPS3r, V-type H^+^ translocating ATPase. The pH-dependent flux boundaries for these reactions are listed in Supplementary Table S8.

To contextualize the pH adaptation during the pH shift, we adjusted a previously published model^27^ establishing the influence of extracellular pH changes. *E. faecalis* has been shown to maintain a more alkaline intracellular pH when exposed to an acidic environment.^26^ Starting at an extracellular pH of 8, *E. faecalis* maintains a pH gradient across the membrane by increasing the activity of the H^+^ translocating V-type ATPase. The ATPase is the primary mechanism of cellular pH homeostasis^25,26^ and was included in the original model (ATPS3r reaction). We identified three additional pH-dependent processes: an unspecific proton leak depending on the pH gradient,^26^ a proton influx via phosphate transport due to a change in protonation states ($H_{2}{\mathrm{PO}}_{4}^{-}$ to $H^{+}{+\mathrm{HPO}}_{4}^{2-}$ at p*K*_a2_=7.21), and a retention of protons due to a stoichiometry change in L-lactate transport.^29^ On the basis of these processes, new reactions and species specific to the environmental pH were introduced to the genome-scale model (Figure 3, Supplementary Table S7). We also introduced a phosphate sink reaction to account for phosphate uptake rates $\left(0.8\;\mathrm{mmol}\;h^{-1}\;g_{\mathrm{DW}}^{-1}\right)$ that exceeded the steady state requirement of the genome-scale model. The general importance of phosphate uptake has been studied for other lactic acid bacteria.^30^ Although teichoic acids and other phosphorylated compounds might have a role as potential phosphate storage, no detailed compositional information exists to our knowledge. We estimated the rate of the proton leak based on membrane conductance^26^ and the contribution of proteins to the dry weight (40%).^31^ Flux boundaries for all pH-dependent reactions are summarized in Supplementary Table S8.

### Integration of proteomic data into the genome-scale model

To integrate the proteomic data into the genome-scale model, we first inactivated 194 proteins (Supplementary Table S4; 29%) coding for 83 reactions (12%) that were not detected during proteomic analysis. The corresponding reactions were switched off if no alternative active configuration was possible based on the annotated gene–protein reaction association (Materials and Methods) and the detected proteins.

Inactivation of undetected proteins created additional essentialities in the genome-scale model as we limited its ability to compensate for inactivated reactions. Therefore, 15 selected proteins were reactivated to ensure a feasible solution that fits the growth parameters of the chemostat culture (Supplementary Table S9). Except for the cystathione g-lyase (EF3284), fructose-6-phosphate aldolase (EF3304), and thiamine diphosphokinase (EF3117), all reactivated proteins are associated to the membrane and, as discussed above, might be missing from the proteomic data set due to their poor solubility. Using this amended model configuration, we calculated the flux distribution for pH 7.5 using FVA.

To ensure that the number of reactivated proteins does not exceed an acceptable limit, we estimated the probability of a protein being present in the sample and going undetected $P\left({\mathrm{protein}}_{\mathrm{expressed}}{|\mathrm{protein}}_{\mathrm{undetected}}\right)$. Given the characteristics of OpenSwath,^32^ which was used to analyze the experimental data, we obtained a probability $P\left({\mathrm{protein}}_{\mathrm{expressed}}{|\mathrm{protein}}_{\mathrm{undetected}}\right)$ in the range of 15–19% (Supplementary Information, section 2). In the model this corresponds to $\frac{{\mathrm{proteins}}_{\mathrm{reactivated}}+\mathrm{essential}\;{\mathrm{proteins}}_{\mathrm{missing}}}{{\mathrm{proteins}}_{\mathrm{inactivated}}+\mathrm{essential}\;{\mathrm{proteins}}_{\mathrm{missing}}}=\frac{15+19}{194+19}=0.16$, which is well within the boundaries set by the analysis method.

To further constrain the genome-scale model, we determined significant protein concentration changes throughout the pH shift experiment using a Bonferroni corrected *P* value threshold of *P*_*i*_*<*0.05 and applied these as additional flux boundaries during FVA such that flux bounds_new_=flux bounds_old_×(fold change±change tolerance). Here we assumed that changes in protein concentration equal changes in catalytic activity. To allow for variations in enzyme activity due to regulatory effects, we included a tolerance of 40% (Supplementary Figure S4). To ensure that this tolerance did not introduce a bias we repeated our computations with tolerances of 20, 60 and 80% with the result that neither 20 nor 60% tolerance changed optimal solution and intervals of FVA. However, a tolerance of 20% demanded additional adjustment of one reaction. With 80%, the optimal solution was almost identical, but intervals of FVA increased.

**Figure 4:**
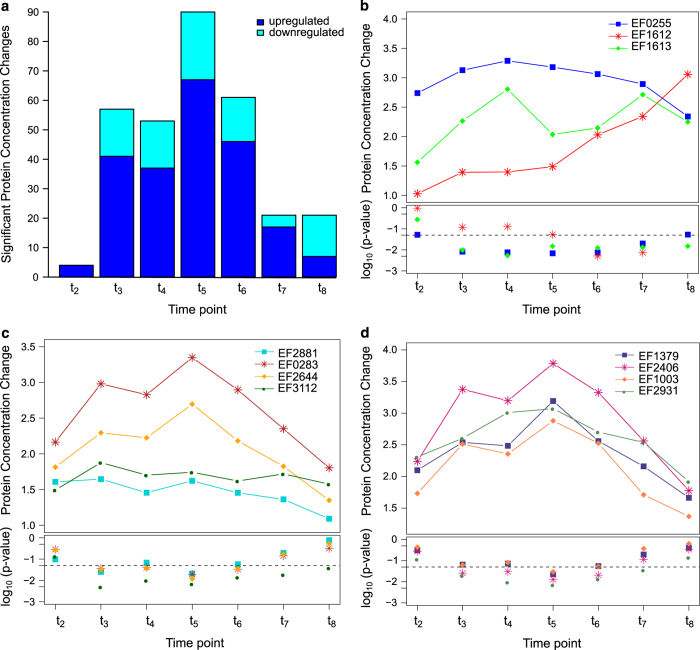
Significant protein concentration changes in response to the pH shift (pH 7.5 *t*_1_, pH 7 *t*_2_, pH 6.5 *t*_3−8_). (**a**) Significant protein concentration changes per time point. Only proteins which are part of the model are shown. Upregulated reactions are indicated in blue, downregulated reactions in cyan. A list of the significant changes of time point *t*_8_ can be found in Table 1. A comprehensive list of all significant protein quantity changes for all time points can be found in the appendix Supplementary Table S10. (**b**) Increased expression of Rex-regulated proteins with respective *P* values. EF0255: L-lactate dehydrogenase (LDH1), EF1612: pyruvate formate-lyase activating enzyme, EF1613: pyruvate formate-lyase (PFL). (**c**) Cell membrane lipoteichoic acid precursor synthesis proteins. EF2881: [acyl-carrier-protein]:NADP^+^ oxidoreductase, EF0283: malonyl-[acyl-carrier-protein] C-acyltransferase, EF2644: phosphoglycerate mutase family protein, EF3112: phosphate acyltransferase. (**d**) Upregulated tRNA producing enzymes. EF1379: alanyl-tRNA synthetase, EF2406: glycyl-tRNA synthetase, EF1003: isoleucyl-tRNA synthetase, EF2931: valyl-tRNA synthetase. tRNA, transfer RNA.

By changing both flux bounds concomitantly, we ensured recursive behavior for subsequent decreasing and increasing protein concentrations (and vice versa). In the experimental data, we observed the majority of significant protein concentration changes within 80 to 180 min after initiating the pH shift (Figure 4a). After 21 h (steady-state culture at pH 6.5, *t*_8_) only 21 significant protein concentration changes remained (Table 1), 7 increasing and 14 decreasing. We were able to apply the changes of 20 of these 21 proteins to the model as additional flux bounds. The remaining protein, a phosphate acyltansferase (EF3112), responsible for the reaction R_GAT1_EFA is essential and produces lipoteichoic acid precursors which feed directly into the objective function. As the composition of the *Lactobacillus plantarum*-derived^33^ biomass reaction is not subjected to pH-dependent changes in the model and the significant protein concentration change thereof cannot be applied to the model. The corresponding model reactions of another 13 proteins are associated with a model flux of zero. Thus, applying the protein concentration change did not affect the flux through these reactions. The decreased protein concentration of aspartate kinase (EF0368) and glutamate dehydrogenase (EF1415) had to be augmented assuming that neither enzyme was working at maximum capacity for the model to have a feasible solution with the existing metabolic and proteomic constraints.

We also observed an upregulation of enzymes in both fermentation branches (Figure 4b): LDH1 (L-lactate dehydrogenase 1, EF0255) and PFL (pyruvate formate-lyase, EF1613) as well as PFL activating enzyme (EF1612). Although the level of LDH1 upregulation falls below the significance level (*P* value: 0.053), both enzymes are regulated by the Rex transcription factor EF2638 in a NADH-responsive manner^34^ prompting us to consider the change in LDH1 levels. In combination with the metabolic data, where we observed a shift from mixed acid to homolactic fermentation, the data suggest that an additional regulatory mechanism on the level of individual enzyme activities is likely to be in effect.

During the transient phase of the pH shift experiment we observe a number of significant changes affecting the synthesis of cell membrane and wall components (Figure 4c) and transfer RNA(tRNA) production (Figure 4d). Both were induced during early pH shift (*t*_2_) and reach a significant increase 80 to 120 min after initiating the pH shift. The latter include enzymes required for biosynthesis of particular tRNA molecules, including those for L-alanine (EF1379), glycine (EF2406), L-isoleucine (EF1003) and L-valine (EF2931) and were upregulated (Figure 4d). These amino acids are found predominantly in transmembrane segments of membrane proteins.^35^ Altogether, this implies a restructuring of the membrane affecting the fatty acid composition, membrane proteins and also lipoteichoic acids in the cell wall as a way to adapt to a more acidic environmental pH.

### Contextualizing pH adaptation of *E. faecalis* in the genome-scale model

To contextualize both proteomic and metabolic adaptive changes we analyzed changes in flux distributions between pH 7.5 and 6.5 using the amended genome-scale model containing pH-specific environmental constraints, experimentally determined metabolite fluxes, and both detected proteins and significant concentration changes thereof. Using this model, we were able to predict the growth rates for both pH 7.5 and 6.5 correctly. In general, we observed that integrating the proteome data lead to smaller intervals for FVA and to a reduction of unconstrained reactions. The model also predicts an increase in the flux through the ATPS3r (*H*^+^ translocating V-type ATPase) reaction by 36% from 1.65 to $2.24\;\mathrm{mmol}\;h^{-1}g_{\mathrm{DW}}^{-1}$, which fits the experimentally observed increase in ATPase activity.^25^

**Figure 5:**
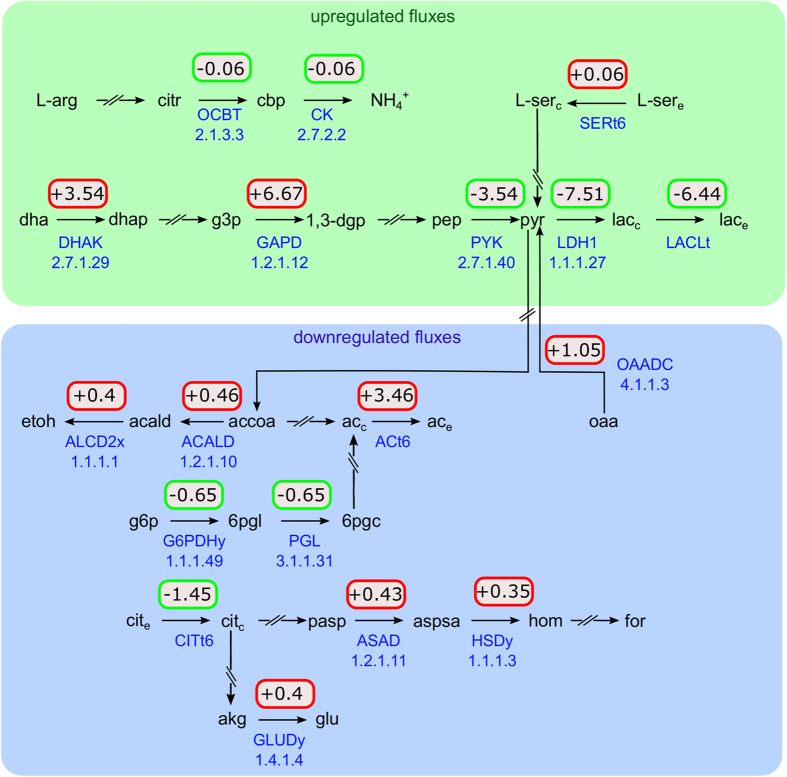
Predicted changes in the flux distributions as computed with FVA between pH 7.5 and 6.5 involving protons. Upregulated fluxes (green) include the main glycolytic pathway, lactate production and degradation of L-arginine and L-serine, downregulated ones (blue) comprise fermentative reaction, the pentose-phosphate pathway (PGL) as well as one reaction from the amino acid pathway (GLUDy). Reactions not involving protons are omitted. The change in proton production per reaction is given in $\mathrm{mmol}\;g_{\mathrm{DW}}^{-1}\;h^{-1}$. ACt6, acetate transport in/out via proton symport; ASAD, aspartate-semialdehyde dehydrogenase; CITt6, citrate transport in/out via proton symport; CK, carbamate kinase; DHAK, dihydroxyacetone (glycerone) kinase; GAPD, glyceraldehyde-3-phosphate dehydrogenase (NAD); GLUDy, glutamate dehydrogenase (NADP); G6PDHy, glucose 6-phosphate dehydrogenase, HSDy, homoserine dehydrogenase (NADPH); OAADC, oxaloacetate decarboxylase; OCBT, ornithine carbamoyltransferase; PGL, 6-phosphogluconolactonase; SERt6, L-serine transport in/out via proton symport. The upper and lower bounds as well as the flux interval size are shown in Supplementary Figure S5.

Interestingly, even though there is an increased glycolytic flux at pH 6.5, the model predicts a reduced proton production by $3.54\;\mathrm{mmol}\;h^{-1}\;g_{\mathrm{DW}}^{-1}$ due to the shift from mixed acid to homolactic fermentation (Figure 5), the less effective lactate transport at pH 6.5 notwithstanding. Without this adaptation, an additional $1.77\;\mathrm{mmol}\;h^{-1}\;g_{\mathrm{DW}}^{-1}$ ATP would need to be invested into the ATPS3r reaction to compensate for the additional protons. Thus, the shift to homolactic fermentation represents a significant cost reduction with respects to intracellular pH homeostasis.

Furthermore, we observed an increased uptake of L-arginine and L-serine. In the model, this translates into an increased ATP production. Although the degradation of L-arginine via citrulline and carbamylphosphate consumes protons, we exclude the possibility of a buffering effect by ${\mathrm{NH}}_{3}{/\mathrm{NH}}_{4}^{+}$ in either extracellular medium (pH 7.5 to 6.5) or cytoplasm (pH 7.9 to 7.6) due to the acidity of ammonia (p*K*_*a*_=9.25).

## Discussion

This study represents the first effort of its kind to combine genome-scale modeling with whole-cell quantitative proteomic data and metabolic data. Previous efforts to integrate proteomic data with genome-scale models have been limited to integrating absolute quantitative data of core proteins,^36^ inactivating undetected proteins,^19,37^ or integrating significant quantity changes in the form of binary states.^10,20^

Here we developed a data integration method and combined quantitative whole-cell proteomic data with genome-scale modeling to study the response of the human pathogen *E. faecalis* to a more acidic environment as in its native environment, the human gastrointestinal tract.^38^ The modeling was based on the validated genome-scale model of *E. faecalis*.^27^ We first integrated experimentally determined metabolite measurements and adjusted the model to include pH-gradient dependent processes to mimic changing extra- and intracellular pH changes. Second, we reduced the genome-scale model to a physiological state where all proteins that are absent at certain environmental conditions are switched off. Third, we integrated quantitative protein changes as additional constraints on flux bounds of the corresponding model reactions. In contrast to applied user-supplied thresholds to derive ‘on’ and ‘off’ states,^9^ we retained the quantitative information from the proteomic data to impose additional flux constraints. Both strategies are based on the assumption that the presence of a protein implies its unconditional activity. As this is not universally true, a few mismatches are expected and can even indicate new regulatory mechanisms on a post-translational or kinetic level.

Strictly speaking, quantitative proteomic data can only be used to define the upper bound of the flux through any metabolic reaction, where the maximal capacity of metabolic flux is determined by the enzyme concentration and the turnover rate of the enzyme. The same principle applies to the integration of transcriptomic data: In PROM^6^ only the upper flux bound of a reaction is changed by calculating *P*×*V*_max_, where *P* is the probability of a gene being expressed, based on gene expression data, and *V*_max_ is the maximal flux as estimated by FVA. Similar to our integration strategy and the one employed by Shlomi *et al.*,^4^ PROM also includes a tolerance to the changed flux bound to account for regulatory mechanisms affecting enzyme activity. Thus, we altered both upper and lower flux bound in our approach and used a global weighting factor to account for enzymes not working at maximal capacity or being restricted by regulatory effects in order to ensure recursive behavior when applying quantitative changes.

A number of reactions did not carry a flux at pH 7.5 and, thus, were not affected by the application of the significant fold changes. Several of these reactions are involved in the *de novo* synthesis of purine nucleotides (R_PRFGS, R_IMPC, R_ADSS, R_AICART). As the medium includes precursors for these, the reactions do not carry a flux in the model. In fact, as FBA and FVA aim for the most efficient solution and the *de novo* synthesis of purine nucleotides is energetically less efficient, no flux in these reactions is to be expected.^39^ In other lactic acid bacteria, the expression of enzymes involved in the *de novo* purine synthesis is regulated by the PurR repressor,^40^ which is activated by purine hypoxanthine and PRPP. *E. faecalis* itself has such a PurR repressor (EF0058) with high sequence identity^27^ to the repressors of *Bacillus subtilis*^41^ and *Lactococcus lactis*.^40,42^ This suggests that the increased availability of purine precursors at pH 6.5 due to the decreased biomass causes a stronger repression of the genes involved in the *de novo* synthesis of purine nucleotides, which we see in the experimental data. Similarly, the enzyme glycerol kinase (R_GLYK) showed a significant decrease in protein levels but did not carry any metabolic flux in the genome-scale model. In *Enterococcus faecalis* the activity of this enzyme is regulated via phosphorylation by a component of the glycerol-specific PTS system. As glycerol is not present in the growth medium, glycerol is not phosphorylated and no flux through glycerol kinase is expected.^43^ Furthermore, the enyzme ribosylhomocysteinase (R_RHC, EF1182), also abbreviated as LuxS, is involved in cell-to-cell signaling,^44^ which cannot be represented using a genome-scale metabolic model, thus, explaining the lack of flux through this reaction. However, the elevated level of RHC in the proteomic data could be explained by the need of cell population to accurately measure cell density in a more competitive environment at pH 6.5.

The amended genome-scale model successfully predicted growth rates of steady-state cultures at pH 7.5 and 6.5. The model showed that a shift to a lower environmental pH strongly increases ATP demand, mainly to maintain the pH gradient across the membrane. In accordance to the increased proton influx into the cell, the flux through the V-type ATPase reaction ATPS3r increases, confirming the experimental measurements of an increase in proton pump activity.^25^ The increasing ATP requirement causes a decrease in biomass and different metabolic adaptations, such as increased glucose, L-arginine and L-serine consumption, and a shift in fermentation favoring homolactic fermentation. The change in fermentation products reduces the amount of protons produced in the central metabolism representing a significant cost reduction with respect to intracellular pH homeostasis. Owing to the pH ranges in cytoplasm and extracellular medium we exclude the possibility of a buffering effect by ${\mathrm{NH}}_{3}{/\mathrm{NH}}_{4}^{+}$.

In combination with the proteomic data, where we observed a concomitant increase of PFL and LDH1, we suggest the existence of a regulatory mechanism on the level of enzyme activity independent of intracellular pH. As the change in intracellular pH within the observed external pH shift is minimal^25,26^ it cannot affect enzyme activity to such a high degree.^45^ An alternative potential regulatory mechanism has been shown to exist in *Lactococcus lactis* where LDH is regulated by the NAD^+^/NADH ratio, which is determined by the glycolytic flux.^46^ An increase in glucose consumption results in a higher NADH concentration activating LDH and inhibiting glyceraldehyde-3-phosphate dehydrogenase. In combination with the inhibition of PFL by glyceraldehyde-3-phosphate, this results in an increased lactate production. Although this regulatory mechanism has yet to be observed for *E. faecalis*, it would explain the metabolic data in context of the proteome measurement.

Furthermore, we observed transient increases of proteins involved in the synthesis of fatty acids and cell wall components. For other lactic acid bacteria, *Streptococcus salvarius* and *Lactobacillus casei*, a change in membrane composition in response to decreasing environmental pH has been reported^47^ to decrease membrane permeability with respect to protons. Thus, we propose that *E. faecalis* reduces its susceptibility to the pH gradient. Furthermore, the change in teichoic acid precursor synthesis remains elevated even after 21 h, suggesting a sustained change in cell wall composition to reduce proton leak into the cell.

In conclusion, we see that proteomic data can be understood and analyzed with the model as a representation of the metabolic network in a very comprehensive, systemic way. However, we also see that proteomic data do not eliminate the need for metabolite measurements because of the differences between proteomic and metabolic changes regarding the behavior of L-lactate/LDH1 and formate/PFL. In fact, the difference itself represents an opportunity to discern regulatory mechanisms and aspects for further studies. Finally, we conclude that the integration of proteomic data into genome-scale models aids in constraining the model in order to represent a more realistic picture of the organism’s state, and thereby limiting the often large solution space.

## Materials and methods

### Bacterial strain and culture conditions

See Supplementary Information 1.1

### Chemostat pH shift experiments

See Supplementary Information 1.2

### Sampling for downstream analyses

See Supplementary Information 1.3

### Proteome sample preparation

See Supplementary Information 1.4

### Sample preparation for SWATH assay library generation

See Supplementary Information 1.5

### Shotgun MS for spectral library generation

See Supplementary Information 1.6

### Spectral and assay library generation

See Supplementary Information 1.7

### DIA mass spectrometry (SWATH-MS)

See Supplementary Information 1.8

### SWATH-MS targeted data extraction

See Supplementary Information 1.9

### Data processing using MSstats

See Supplementary Information 1.10

### Genome-scale metabolic model of *Enterococcus faecalis*

See Supplementary Information 1.11

### Proteome coverage of the genome-scale model

See Supplementary Information 1.12

### Introducing gene-protein-reaction associations

See Supplementary Information 1.13

### Reaction and gene essentiality scan

See Supplementary Information 1.14

### Model simulation

See Supplementary Information 1.15

## Figures and Tables

**Table 1:** Reactions with significant quantity changes on protein level between time points *t*_1_ and *t*_8_

*Reaction ID*	*Name*	*Protein ID*	*Change*	P-*value*	*EC number*
R_ACTPASE	Acylphosphatase	EF2401	0.452	0.0013	3.6.1.7
R_ADPT	Adenine phosphoribosyltransferase	EF1687	1.6662	0.0149	2.4.2.7
R_ADSS	Adenylosuccinate synthetase	EF0014	0.5674	0.0075	6.3.4.4
R_AICART	Phosphoribosylaminoimidazolecarboxamide formyltransferase	EF1778	0.41	0.0421	2.1.2.3
R_ASPK	Aspartate kinase	EF0368	0.4923	0.0202	2.7.2.4
R_CDD	Cytidine deaminase	EF0175	0.4768	0.0001	3.5.4.5
R_GAT1_EFA	Glycerol 3-phosphate acyltransferase	EF3112	1.5759	0.0379	2.3.1.15
R_GLUDy	Glutamate dehydrogenase	EF1415	0.609	0.0395	1.4.1.4
R_GLYK	Glycerol kinase	EF1929	0.5845	0.0448	2.7.1.30
R_IMPC	IMP cyclohydrolase	EF1778	0.41	0.0421	3.5.4.10
R_LDH1^a^	Lactate dehydrogenase	EF0255	2.3418	0.0531	1.1.1.27
R_PDE1	3′5′-cyclic-nucleotide phosphodiesterase	EF0063	0.4704	0.0028	3.1.4.17
R_PDE2	3′5′-cyclic-nucleotide phosphodiesterase	EF0063	0.4704	0.0028	3.1.4.17
R_PDE3	3′5′-cyclic-nucleotide phosphodiesterase	EF0063	0.4704	0.0028	3.1.4.17
R_PDE4	3′5′-cyclic-nucleotide phosphodiesterase	EF0063	0.4704	0.0028	3.1.4.17
R_PDE5	3′5′-cyclic-nucleotide phosphodiesterase	EF0063	0.4704	0.0028	3.1.4.17
R_PFL	Formate C-acetyltransferase	EF1612	3.057	0.0001	2.3.1.54
R_PGL	6-Phosphogluconolactonase	EF1918	1.7277	0.0092	3.1.1.31
R_PPM2	Phosphopentomutase (deoxyribose)	EF0185	2.046	0.0156	5.4.2.7
R_PRFGS	Phosphoribosylformylglycinamidine synthase	EF1784	0.1896	0.0028	6.3.5.3
R_RHC	Ribosylhomocysteinase	EF1182	1.7792	0.0319	4.2.1.21
R_UDPG4E	UDPglucose 4-epimerase	EF1070	2.3293	0.0109	5.1.3.2

Abbreviation: EC, Enzyme Commission.

a Lactate dehydrogenase missed the threshold by 0.03 but was included due to the metabolic data. A comprehensive list of the significant protein quantity changes for all time points can be found in the Supplementary Table S10.
