# Supplementary material for: Integrating highly quantitative proteomics and genome-scale metabolic modeling to study pH adaptation in the human pathogen Enterococcus faecalis
Source: NPJ Syst Biol Appl. 2016 Sep 8;2:16017–. doi: 10.1038/npjsba.2016.17 (PMC5516852; doi:10.1038/npjsba.2016.17)
Supplement: Supplementary Information [file npjsba201617-s1.doc]

Supplements

Contents

[1 Supplementary methods 2](#__RefHeading___Toc445103128)

[1.1 Bacterial strains and culture conditions 2](#__RefHeading___Toc445103129)

[1.2 Chemostat pH shift experiments 2](#__RefHeading___Toc445103130)

[1.3 Sampling for downstream analyses 3](#__RefHeading___Toc445103131)

[1.4 Proteome sample preparation 3](#__RefHeading___Toc445103132)

[1.5 Sample preparation for SWATH assay library generation 4](#__RefHeading___Toc445103133)

[1.6 Shotgun MS for spectral library generation 5](#__RefHeading___Toc445103134)

[1.7 Spectral and assay library generation 5](#__RefHeading___Toc445103135)

[1.8 DIA mass spectrometry (SWATH-MS) 6](#__RefHeading___Toc445103136)

[1.9 SWATH-MS targeted data extraction 6](#__RefHeading___Toc445103137)

[1.10 Data processing using MSstats 7](#__RefHeading___Toc445103138)

[1.11 Genome-scale metabolic model of *Enterococcus faecalis* 8](#__RefHeading___Toc445103139)

[1.12 Proteome coverage of the genome-scale model 8](#__RefHeading___Toc445103140)

[1.13 Introducing gene-protein-reaction associations (GPRs) 10](#__RefHeading___Toc445103141)

[1.14 Reaction and gene essentiality scan 10](#__RefHeading___Toc445103142)

[1.15 Model simulation 10](#__RefHeading___Toc445103143)

[2 Supplementary figures 13](#__RefHeading___Toc445103144)

[3 Supplementary tables 17](#__RefHeading___Toc445103145)

[4 Proteome Integration 39](#__RefHeading___Toc445103146)

[5 Supplementary references 44](#__RefHeading___Toc445103147)

# Supplementary methods

## Bacterial strains and culture conditions

*Enterococcus faecalis* V583 wild type was grown in batch cultures at 37 °C in a chemically defined medium for lactic acid bacteria (CDM-LAB, pH 7.4). The CDM-LAB medium^1^ contained the following per liter: 1 g K_2_HPO_4_, 5 g KH_2_PO_4_, NaHCO_3_, 0.6 g ammonium citrate, 1 g acetate, 0.25 g tyrosine,0.24 g alanine, 0.125 g arginine, 0.42 g aspartic acid, 0.13 g cysteine, 0.5 g glutamic acid, 0.15 g histidine, 0.21 g isoleucine, 0.475 g leucine, 0.44 g lysine, 0.275 g phenylalanine, 0.675 g proline, 0.34 g serine, 0.225 g threonine, 0.05 g tryptophan, 0.325 g valine, 0.175 g glycine, 0.125 g methionine, 0.1 g asparagine, 0.2 g glutamine, 10 g glucose, 0.5 g L-ascorbic acid, 35 mg adenine sulfate, 27 mg guanine, 22 mg uracil, 50 mg cystine, 50 mg xanthine, 2.5 mg D-biotin, 1 mg vitamin B12, 1 mg riboflavin, 5 mg pyridoxamine-HCl, 10 mg p-aminobenzoicacid, 1 mg pantothenate, 5 mg inosine, 1 mg nicotinic acid, 5 mg orotic acid, 2 mg pyridoxine, 1 mg thiamine, 2.5 mg lipoic acid, 5 mg thymidine, 200 mg MgCl_2_, 50 mg CaCl_2_, 16 mg MnCl_2_, 3 mg FeCl_3_, 5 mg FeCl_2_, 5 mg ZnSO_4_, 2.5 mg CoSO_4_, 2.5 mg CuSO_4_, and 2.5 mg (NH4)_6_Mo_7_O_24_.

## Chemostat pH shift experiments

*E. faecalis* V583 was grown in an anaerobic glucose limited chemostat culture in CDM-LAB medium as described previously^1,2^. Cultures were grown in a Biostat Bplus fermenter unit in a total volume of 750 ml at a stirring rate of 200 RPM, and under constant gassing with 50 ml/min nitrogen. The temperature was kept at 37 °C. The pH was maintained at the indicated value by titrating with sterile 3 M *KOH*. In a standard experiment, the culture vessel with 750 ml CDM-LAB (pH 7.5) was inoculated with 20 ml of an *E. faecalis* over-night culture in CDM-LAB. Bacteria were allowed to grow for 2 h before medium flow-through was started. Growth rates were controlled by the medium dilution rate (
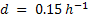
). Culture volume was kept constant by removing culture liquid with the same rate as fresh medium was added. The pH was kept constant at pH 7.5 until cultures reached a steady state. Steady state was assumed when no detectable glucose remained in the culture supernatant and optical densities, dry weights, and product concentrations of the cultures were constant on two consecutive days. For pH shift experiments, steady state was established at pH 7.5 and then pH control was switched off. The pH value was allowed to drop to 6.5 before pH control was reinstated to keep the pH constant at 6.5. Cultivation was continued until steady state at pH 6.5 was reached.

## Sampling for downstream analyses

For determination of cell dry weight, cells of 10 ml culture per biological replicate per sampling point were pelleted by centrifugation, washed 3 times with 1xPBS (pH 7) and freeze-dried. For the proteome analysis, another 20 ml of culture were centrifuged and cell pellets were treated as described above. Dried pellets were stored at -80 °C. Quantification of amino acids was done by Frank Gutjahr Chromatographie (Balingen, Germany) from 2 ml of filter-sterilized culture supernatant. Concentrations of lactate, formate, acetate, glucose, acetoin, 2,3-butanediol, ascorbate, citrate, pyruvate and ethanol were determined from 5-10 ml filter-sterilized culture supernatant by Metabolomics Discoveries GmbH (Potsdam, Germany).

## Proteome sample preparation

The *E. faecalis* V583 bacterial cell pellets were harvested, washed 3 times with PBS and were kept frozen until experimentation began. These non-viable cell pellets were processed in two technical replicates using BarocyclerrNEP2320 (PressureBioSciences, Inc, South Easton, MA). Briefly, samples were lysed in buffer containing 8 M urea, 0.1 M ammonium bicarbonate, 10% trifluoroethanol, and completeTM protease inhibitor under pressure cycling (PCT) program (198 cycles, 20 seconds 45 kpsi, 10 seconds 0 kpsi) at 35 °C. Whole cell lysates were then sonicated for 30 seconds with 1-minute interval on ice for 3 times. Cellular debris was removed by centrifugation and sample protein concentration was determined by BCA assay prior to protein reduction with 10 mM TCEP for 25 min at 35 °C, and alkylation with 40 mM iodoacetamide in the dark for 30 min at room temperature. LysC digestion (1/50, w/w) was performed in 6 M urea under PCT program: 90 cycles, 25 seconds 22 kpsi, 10 seconds 0 kpsi at 35 °C; subsequent trypsin digestion (1/30, w/w) was performed at further diluted urea (1.6 M) under PCT program: 180 cycles, 25 seconds 22 kpsi, 10 seconds 0 kpsi 35 °C. Digestion was stopped by acidification with trifluoroacetic acid (TFA) to a final pH of approximately 2 before C18 column desalting using SEP-PAK C18 cartridges (Waters Corp., Milford, MA, USA).

## Sample preparation for SWATH assay library generation

To create a comprehensive assay library for SWATH-MS analysis, we pooled peptides from all metabolic conditions - after desalting - from a pilot experiment to perform off-gel fractionation. Pooled peptides were solubilized with OGE buffer containing 5% (v/v) glycerol, 0.7% (v/v) ACN and 1% (v/v) carrier ampholytes mixture (IPG buffer pH 3.0 10.0, GE Healthcare). Fractionation was performed on a 3100 OFFGEL (OGE) Fractionator (Agilent Technologies) using a 24 cm pH3-10 IPG strip (GE Healthcare) according to manufacturer’s instructions using a program of 1 hour rehydration at maximum 500 V, 50 µA and 200 mW followed by the separation at maximum 8000 V, 100 µA and 300 mW until 50 kVh were reached. All 24 fractions were recovered and desalted again by C18 reversed-phase MicroSpin columns (The Nest Group Inc.). Depending on the sample complexity, neighbouring fractions were pooled into 12 samples for subsequent analysis by mass spectrometry: pool 1 (fraction 1-2), pool 2 (fraction 3), pool 3 (fraction 4), pool 4 (fraction 5), pool 5 (fraction 6-7), pool 6 (fraction 8-9), pool 7 (fraction 10-11), pool 8 (fraction 12-15), pool 9 (fraction 16-19), pool 10 (fraction 20-21), pool 11 (fraction 22), pool 12 (fraction 23-24).

## Shotgun MS for spectral library generation

For spectral library generation, an AB SCIEX TripleTOF 5600 mass spectrometer was operated in data/information-dependent acquisition (IDA) mode, essentially as previously described^3^: all samples were analyzed on an Eksigent nanoLC (AS-2/1Dplus or AS-2/2Dplus) system coupled with a SWATH-MS-enabled AB SCIEX TripleTOF 5600 System. The HPLC solvent system consisted of buffer A (2% acetonitrile and 0.1% formic acid, v/v) and buffer B (95% acetonitrile with 0.1% formic acid, v/v). Samples were separated on a 75 µm - diameter PicoTip emitter (New Objective) packed with 20 cm of Magic 3 µm, 200A C18 AQ material (Bischoff Chromatography). The loaded material was eluted from the column at a flow rate of 300 nl/min with the following gradient: linear 2 - 35% B over 120 min, linear 35 - 90% B for 1 min, isocratic 90% B for 4 min, linear 90 2% B for 1 min and isocratic 2% solvent B for 9 min. The mass spectrometer was operated in DDA top20 mode, with 500 ms and 150 ms acquisition time for the MS1 and MS2 scans respectively, and 20 s dynamic exclusion. Rolling collision energy with a collision energy spread of 15 V was used for fragmentation.

## Spectral and assay library generation

All raw instrument data were centroided using Proteowizard msconvert (2.0). The assay library was generated using an established protocol^4^: The Trans-Proteomic Pipeline (TPP)^5^ (4.7.0) and SpectraST^6^ (5.0) were used to analyze the shotgun (DDA) datasets. The data sets were searched individually using X!Tandem ^7^ (2011.12.01.1) with k-score plugin^8^, Mascot 2.4 and Comet^9^ (2013.02r2) against the reference proteome of *Enterococcus faecalis* (strain ATCC 700802 / V583) annotated in UniProtKB/SwissProt (2014_02) and appended with iRT peptide (Escher *et al.*, 2012) and decoy sequences for retention time alignment and error rate estimation. Carbamidomethyl (C) was used as a fixed modification; Oxidation (M), the only variable modification. Two peptide missed cleavages were allowed. Parent mass error was set to ±50 ppm, fragment mass error to ±0.1 Da. The search identifications were combined and statistically scored using PeptideProphet^10^ and iProphet^11^ available within the TPP toolset. MAYU^12^ (1.07) was used to control for peptide FDR at 1% (corresponding to protein FDR 4%) with an iProphet cutoff of 0.915593. SpectraST was used in library generation mode with CID-QTOF settings and iRT normalization at import against the iRT Kit peptide sequences (-c_IRTirt.txt -c_IRR), where the empirical retention times were transformed into the normalized iRT space using a linear regression with the iRT peptides^13^, and a consensus library was consecutively generated^14^. The script spectrast2tsv.py^4^ (msproteomicstools 0.2.2) was then used to generate the assay library with the following settings: : -l 350,2000 -s y,b -g -18.010565, -17.026549, -63.998287, -79.966333, -97.976898 -x 1,2,3,4 -o 6 -n 6 -p 0.05 -e -d -w swaths_64w.txt -k openswath. The OpenSWATH tool, ConvertTSVToTraML converted the TSV file to TraML format; OpenSwathDecoyGenerator generated the decoy assays in shuffle mode and appended them to the TraML assay library for later classification and error rate estimation.

## DIA mass spectrometry (SWATH-MS)

For SWATH-MS data acquisition, the same mass spectrometer and LC-MS/MS setup was operated essentially as described before^3^, except that 64 windows of variable effective isolation widths were used (with an additional 1 Da overlap on the left side of the window), with a dwell time of 100 ms to cover the mass range from 400 - 1200 m/z in 3.3 s. The collision energy for each window was set using the collision energy of a 2^+^ ion centered in the middle of the window with a spread of 15 eV.

## SWATH-MS targeted data extraction

The SWATH targeted data analysis was carried out using (OpenMS 1.12) analysis workflow (OpenSwathWorkflow^15^, http://www.openswath.org) running on an internal computing cluster and consists of the following steps. First, fragment-ion chromatograms were extracted for each peptide precursor in its appropriate SWATH-MS window based on the target and decoy assays in TraML format, with an extraction width of 0.05 Thomson (OpenSwath ChromatogramExtractor) and a retention time extraction window of ±300 seconds around the expected retention time. Additionally, ion chromatograms for the iRT retention time standard peptides were extracted to facilitate projection of the assays from the normalized iRT retention time space into the retention time space for each individual run (OpenSwath RTNormalizer). Peak groups from the extracted fragment-ion chromatograms were formed and scored according to their elution profiles, similarity to the target assay in terms of retention time and relative fragment-ion intensity, as well as features from the full MS2 SWATH spectrum extracted at the chromatographic peak apex (OpenSwath Analyzer). Finally, the optimal separation between true and false peak groups was achieved using a linear discriminant model training with 60-fold semi-supervised learning iterations; and the score distribution from the shuffled decoy assays was used to estimate the false discovery rate using pyProphet (0.9.2) (<https://pypi.python.org/pypi/pyprophet>^15^) based the mProphet algorithm^16^ and filtered using 1% FDR at the peptide feature level. Further, peak-groups were aligned among all 48 SWATH runs using the OpenSwath feature_aligner to ensure the consistent quantification of peak groups (peptide features) that could otherwise not be confidently identified above the FDR cut-off from a single run alone. Re-quantification option was also enabled to provide an upper bound for the intensity of target analyte where no peak-group passed the confidence filter so that the final data matrix did not contain any missing data point.

## Data processing using MSstats

Protein quantification was computed using R package, MSStats.daily 2.3.5^17^. Briefly, we preprocessed the dataset from openSWATH extraction by log2 transformation and quantile normalization and generated the protein quantity matrix from the fragment ion level data using the ’groupComparison’ and ’quantification’ function of MSstats.

The mass spectrometry proteomics data have been deposited to the ProteomeXchange Consortium

(<http://proteomecentral.proteomexchange.org/>) via the PRIDE partner repository with the data set identifier PXD002869. Users can sign in via <http://www.ebi.ac.uk/pride/archive/> to access the SWATH data with:

Reviewer account details:

Username: [reviewer83899@ebi.ac.uk](mailto:reviewer83899@ebi.ac.uk)

Password: 3FehKjHM

For the shotgun/ DDA mass spectrometry data:

Dataset Identifier: PXD001576

Username: [reviewer49016@ebi.ac.uk](mailto:reviewer49016@ebi.ac.uk)

Password: khGEQS3G

## Genome-scale metabolic model of *Enterococcus faecalis*

The manually curated and validated genome-scale metabolic model, originally published in 2015 by Veith *et al.*^18^, contains 642 metabolites and 706 reactions. The genome-scale model was validated using predictions of amino acid auxotrophies, all of which were verified experimentally. The model has been used to study the amino acid metabolism of *Enterococcus faecalis* and the phenotype of a glutamine synthetase knockout mutant.

## Proteome coverage of the genome-scale model

To investigate the causes of the observed metabolite changes we sampled intracellular proteins of *E. faecalis* throughout the entire time-course and analyzed detected proteins and significant protein concentration changes. The protein samples were analyzed by SWATH-MS. This mass spectrometry-based technique relies on an organism-specific assay library that has to be developed prior to the actual experiment and then serves as the basis for the detection and quantification of query proteins in the samples^4^. Our assay library covers 2282 (70%) of the 3240 annotated *E. faecalis* proteins in UniProtKB andallowed us to quantify 1717 (53%) proteins consistently across all samples at 4% FDR (false discovery rate) with the openSWATH software tool^15^ (Fig S1A and B). This number likely approximates the number of proteins that is actually expressed by *E. faecalis* under the study conditions with the exception of insoluble membrane proteins. Membrane-associated proteins contain highly hydrophobic inter-membrane regions or anchor domains, which lower their solubility and hampered their extraction. These proteins are therefore more likely to be under-represented in the proteomic data. Of all identified proteins, we considered 1,681 proteins for subsequent integration and analysis. Overall, we observed a strong correlation between biological and technical replicates for all time points (
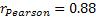
, Fig S2).

To assess the suitability of the proteomic data for integration into the model, we compared the detected proteins to characteristic properties such as proteins predicted to be essential by the model. Essential proteins have to remain active for the model to have a feasible solution and as such were exempted during the inactivation of undetected proteins. The genome-scale model of *E. faecalis*^18^ contains a total of 709 reactions, of which 170 (24%) are essential (Fig S3A, Tab S1), and 668 proteins, of which 101 (15%) are essential (Fig S3B, Tab S2).

As annotated in the model, 18 essential proteins were missing in the proteomic data (Tab S3). Most of these proteins are associated with the membrane and include proteins like the transporters for nicotinic acids (EF0739) and biotin (EF3072). Overall, only a small number of proteins predicted to be essential by the model is not covered in the proteomic dataset while over 68% of the entire *E. faecalis* metabolic model is covered.

## Introducing gene-protein-reaction associations (GPRs)

To integrate proteomic data into genome-scale models, we annotated curated gene-protein-reaction associations (GPRs) to the genome-scale model reactions. GPRs link the proteomic data to the genome-scale model by facilitating the mapping process between detected proteins and the respective model reactions. Reactions without an annotated GPR cannot be evaluated using proteomic data and introduce a degree of uncertainty in the model analysis. Using ’AND’ and ’OR’ relationships between individual proteins, we were able to assess the presence of multi-enzyme complexes and isoforms.

## Reaction and gene essentiality scan

We identified essential reactions and proteins by sequentially inactivating individual proteins or model reactions. In the case of reaction scan, the flux boundaries of the evaluated reaction were set to zero. While searching for essential proteins, the protein itself was set inactive and the reaction’s activity was reevaluated. If the reaction could not occur without the inactivated protein, the reaction’s flux bounds were once again set to zero. A protein or reaction was regarded as essential, if the inactivation caused an infeasible FBA solution.

To avoid the prediction of artificial essential reactions and proteins we set the flux bounds of all exchange reactions to
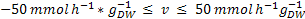
, allowing the model to account for potential changes and adaptations in metabolite profiles.

## Model simulation

Experimentally determined metabolite concentrations are used as model constraints in the form of flux boundaries on the uptake and product fluxes represented by the exchange reactions in the genome-scale models. The flux bounds are calculated by:


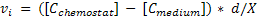


where
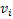
represents the net uptake or product flux, C the metabolite concentration (
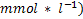
,
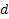
 the dilution rate (
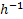
), and
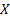
 the dry weight of the cells (
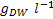
). Medium components not measured experimentally are represented by theoretical rates (
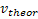
) setting
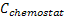
to zero. Flux bounds on the net uptake or product flux were generally defined as
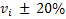
 (based on the average standard deviation of the metabolites at steady state conditions) such that
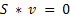
 for all measured fluxes
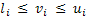
and for
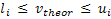
, with
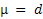
.
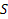
 represents the stoichiometric matrix,
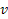
 the flux vector, and
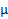
 the growth rate. All measured metabolites including the corresponding flux bounds are listed in the appendix in Tables S5 and S6. Reactions, where the model did not allow for the flux bounds as defined above, are marked accordingly.

Maximal growth was selected as objective function and flux balance analysis (FBA) as well as flux variability analysis (FVA) were used to calculate optimal flux distributions and flux ranges for each flux in the metabolic network, respectively. The modeling was performed with PySCeS CBMpy 0.7.0^19^ (http://cbmpy.sourceforge.net). The integration of the proteomic data was achieved through the use of custom Python scripting and PySCeS CBMpy.

# Statistical evaluation of the proteomic data integration

During the integration of the proteomic data several proteins were not detected in the data set but were necessary for the model to have a feasible solution. To ascertain that this number of proteins does not exceed an acceptable limit we used the characteristics of the analysis method OpenSwath ^15^ (see section 1.9) to estimate the probability of a protein
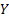
 going undetected even though it is in the sample using Bayesian Theorem:


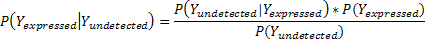


The characteristic of OpenSwath were determined for a false discovery rate (FDR) of 5%, where a recovery rate of 87.5% was obtained. During our analysis we applied a FDR of 4%, which ultimately results in a slight decrease of the recovery rate. We used an assumed recovery rate of 85% as conservative estimate. With 1717 consistently quantified proteins and 3240 annotated proteins in total we obtained:

For a FDR of 5% and 87.5% recovery rate:

|  | 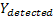 | 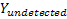 |
| --- | --- | --- |
| 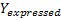 | 1631 | 233 |
| 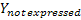 | 86 | 1290 |


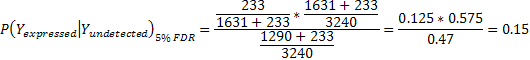


For a FDR of 4% and 85% recovery rate:

|  | 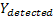 | 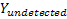 |
| --- | --- | --- |
| 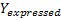 | 1648 | 290 |
| 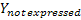 | 69 | 1233 |


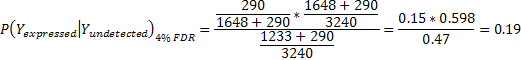


In the model this probability is represented by:


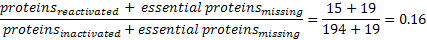


The amount of reactivated proteins is therefore well within the acceptable range determined by the analysis method OpenSwath.

# Supplementary figures

**Figure S1**: *Enterococcus faecalis* V 583 exposed to a pH shift in continuous culture.

**A.** Proteome coverage of *E. faecalis*: of 3240 proteins annotated in UniProtKB, 2282 were detected using mass spectrometry after extensive fractionation (constituting the assay library) and 1717 could be detected consistently in each sample using SWATH-MS.

**B.** The log_10_ abundance of 1790 *E. faecalis* proteins calculated using the ’best flyer peptide’ approach is plotted. Protein abundances of *E. faecalis* as detected by SWATH-MS estimated by the ion count of the most intense peptide.

**Figure S2**: Reproducibility with respect to peptide precursor intensities between technical and biological replicates per time point.

**A**: Biological replicates per time point. Average Pearson correlation coefficient:
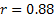
.

**B**: Technical replicates per time point. Average Pearson correlation coefficient:
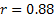
.

**Figure S3**: Essential components in the genome-scale model of *E. faecalis* by metabolic pathway.

**A:** Essential reactions. A list of all essential reactions can be found in Tab S1.

**B:** Essential proteins. A list of all essential proteins can be found in Tab S2.

**Figure S4**: Schematic representation of the application of significant protein quantity changes. Protein quantity changes including a tolerance of ± 40% are applied to both upper and lower flux boundary.


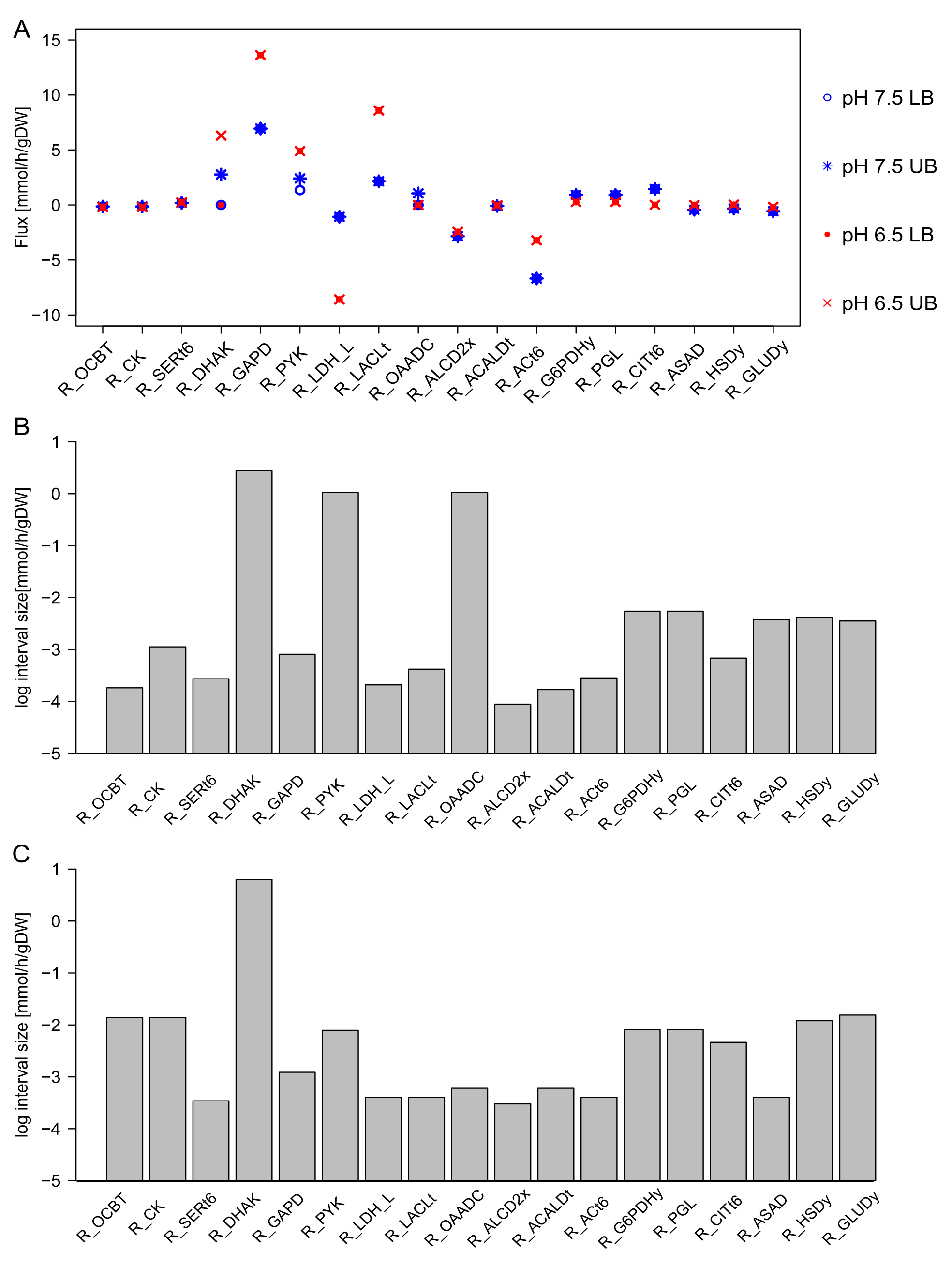


**Figure S5**: FVA results used for Figure 5.

1. Upper and lower flux boundaries for pH 7.5 (blue) and pH 6.5 (red).
2. Logarithmic flux interval size for pH 7.5.
3. Logarithmic flux interval size for pH 6.5.

# Supplementary tables

**Table S1** Essential reactions.

| ID | Reaction | E.C. # |
| --- | --- | --- |
| R_ACACT1 | Acetyl-CoA C-acetyltransferase | 2.3.1.9 |
| R_ACCOAC | acetyl CoA carboxylase | 6.4.1.2 |
| R_AGAT_EFA | 1Acyl-glycerol-3-phosphate acyltransferase | 2.3.1.51 |
| R_ALAR | alanine racemase | 5.1.1.1 |
| R_ALATA_Lr | alanine transaminase | 2.6.1.2 |
| R_ALATRS | ala tRNA synthetase | 6.1.1.7 |
| R_ALKP_Efa | alkaline phosphatase | 3.1.3.1 |
| R_ARGTRS | arg tRNA synthetase | 6.1.1.19 |
| R_ASPTRS | asp tRNA synthetase | 6.1.1.19 |
| R_BIOMASS | biomass reaction |  |
| R_BPPA1 | UDP-N-acetyl-muramoylpentapeptide-lysine-N6-alanyltransferase | 2.3.2.- |
| R_BPPA1_L | UDP-N-acetyl-muramoylpentapeptide-lysine-N6-alanyltransferase | 2.3.2.- |
| R_BPPA2 | UDP-N-acetyl-muramoylpentapeptide-lysine-N6-alanyltransferase | 2.3.2.- |
| R_BPPA2_L | UDP-N-acetyl-muramoylpentapeptide-lysine-N6-alanyltransferase | 2.3.2.- |
| R_BTMAT1 | Butyryl-[acyl-carrier protein]:malonyl-CoA acyltransferase |  |
| R_BTNt2i | Biotin uptake |  |
| R_CLPNS_LPL | cardiolipin synthase | 2.7.8.- |
| R_CPS_EFA_SYNTH | synthesis of capsule and wall PS |  |
| R_CPS_PS_EFA | Capsule-polysaccharide EFA presumably cps gene cluster |  |
| R_CRCT | CTP:D-ribitol-5-phosphate citidylyltransferase |  |
| R_CYSTRS | cys tRNA synthetase | 6.1.1.16 |
| R_CYTK1 | citidylate kinase | 2.7.1.48 |
| R_DAGGT_LPL | 1,2-diacylglycerol-3-glucosyltransferase | 2.4.1.157 |
| R_DAGK_LPL | diacylglycerol kinase | 2.7.1.107 |
| R_DALTAL_EFA | D-alanine lipoteichoic acid ligase | 6.3.2.16 |
| R_DARTAL_EFA | D-alanine lipoteichoic acid ligase |  |
| R_DASYN_LPL | wall teichoic acid modification | 2.7.7.41 |
| R_DDL | D-alanine-D-alanine ligase | 6.3.2.4 |
| R_DDMAT5 | dodecanoly [acyl-carrier protein]: malonyl-CoA C-acetyltransferase | 1.3.1.10 |
| R_DEMAT4 | decanoyl [acyl-carrier protein]:malonyl-CoA C-acyltransferase | 1.3.1.10 |
| R_DEX_PS_EFA | dextran presumable PS in envelope EFA |  |
| R_DHFR | dihydrofolate reductase | 1.5.1.3 |
| R_DHFS | dihydrofolate synthase | 6.3.2.12 |
| R_DHNPA | dihydroneopterin aldolase | 4.1.2.25 |
| R_DHPS3 | dihydropteroate synthase | 2.5.1.15 |
| R_DMATT | dimethylallyltransferase | 2.5.1.1 |
| R_DNAS_LPL | DNA synthesis |  |
| R_DPCOAK | dephospho CoA kinase | 2.7.1.24 |
| R_DPMVD | diphosphomevalonate decarboxylase | 4.1.1.33 |
| R_DTMPK | dTMP kinase | 2.7.4.9 |
| R_EPA_PS_EFA | tetraheteropolysaccharide EFA presumably epa gene cluster |  |
| R_Ex_arg_L__e | arg exchange reaction |  |
| R_Ex_btn__e | btn exchange reaction |  |
| R_Ex_glyclt__e | glyclt exchange reaction |  |
| R_Ex_his_L__e | his exchange reaction |  |
| R_Ex_ile_L__e | ile exchange reaction |  |
| R_Ex_leu_L__e | leu exchange reaction |  |
| R_Ex_nac__e | nac exchange reacton |  |
| R_Ex_pydam__e | pydam exchange reaction |  |
| R_Ex_thm__e | thm exchange reaction |  |
| R_Ex_trp_L__e | trp exchange reaction |  |
| R_Ex_val_L__e | val exchange reaction |  |
| R_FABM1 | fatty acid enoyl isomerase | 5.3.3.14 |
| R_FABM2 | fatty acid enoyl isomerase | 5.3.3.14 |
| R_G1PACT | glucosamine-1-phosphate N-acetyltransferase | 2.3.1.157 |
| R_G1PTMT | glucose-1-phosphate thymidylyltransferase | 2.7.7.24 |
| R_GALU | UTP-glucose-1-phosphate uridylyltransferase | 2.7.7.9 |
| R_GAT1_EFA | glycerol-3-phosphate acyltransferase | 2.3.1.15 |
| R_GCALDD | glycoaldehyde dehydrogenase | 1.2.1.21 |
| R_GLNTAL | glutamyl tRNA:L-glutamine amido ligase | 6.3.5.- |
| R_GLUR | glutamate racemase | 5.1.1.3 |
| R_GLUTRS | glu tRNA synthetase | 6.1.1.17 |
| R_GLYCLTt2r | glycolate transport via proton symport, reversible |  |
| R_GLYTRS | gly tRNA synthetase | 6.1.1.14 |
| R_GRTT | geranyltranstransferase | 2.5.1.10 |
| R_GTPCI | GTP cyclohydrolase I | 3.5.4.16 |
| R_HBUHL1 | (3R)-3-Hydroxybutanoly[acyl-carrier protein] hydro lyase | 4.2.1.58 |
| R_HBUR1 | (3R)-3-Hydroxybutanoly[acyl-carrier protein]: NADP + oxidoreductase | 1.1.1.100 |
| R_HCO3E | carbonate dehydratase | 4.2.1.1 |
| R_HDDHL5 | (3R)-3-Hydroxybutanoly[acyl-carrier protein] hydro lyase | 4.2.1.58 |
| R_HDDR5 | (3R)-3-Hydroxybutanoly[acyl-carrier protein]: NADP + oxidoreductase | 1.1.1.100 |
| R_HDEHL4 | (3R)-3-Hydroxybutanoly[acyl-carrier protein] hydro lyase | 4.2.1.58 |
| R_HDER4 | (3R)-3-Hydroxybutanoly[acyl-carrier protein]: NADP + oxidoreductase | 1.1.1.100 |
| R_HDMAT7 | hexadecanoly-[acyl-carrier protein]:malonyl CoA C acyltransferase | 1.3.1.10 |
| R_HEMAT2 | hexadecanoly-[acyl-carrier protein]:malonyl CoA C acyltransferase | 1.3.1.10 |
| R_HHDHL7 | (3R)-3-Hydroxybutanoly[acyl-carrier protein] hydro lyase | 4.2.1.58 |
| R_HHDR7 | (3R)-3-Hydroxybutanoly[acyl-carrier protein]: NADP + oxidoreductase | 1.1.1.100 |
| R_HHYHL2 | (3R)-3-Hydroxybutanoly[acyl-carrier protein] hydro lyase | 4.2.1.58 |
| R_HHYR2 | (3R)-3-Hydroxybutanoly[acyl-carrier protein]: NADP + oxidoreductase | 1.1.1.100 |
| R_HISt6 | histine transport |  |
| R_HISTRS | his tRNA synthetase | 6.1.1.21 |
| R_HMGCOAR | hydroxymethylglutaryl CoA reductase | 1.1.1.34 |
| R_HMGCOAS | hydroxymethylglutaryl CoA synthase | 2.3.3.10 |
| R_HOCHL3 | (3R)-3-Hydroxybutanoly[acyl-carrier protein] hydro lyase | 4.2.1.58 |
| R_HOCR3 | (3R)-3-Hydroxybutanoly[acyl-carrier protein]: NADP + oxidoreductase | 1.1.1.100 |
| R_HODHL8 | (3R)-3-Hydroxybutanoly[acyl-carrier protein] hydro lyase | 4.2.1.58 |
| R_HODR8 | (3R)-3-Hydroxybutanoly[acyl-carrier protein]: NADP + oxidoreductase | 1.1.1.100 |
| R_HPPK | 2-amino-4-hydroxy-6-hydroxymethyldihydropteridine diphosphokinase | 2.7.6.3 |
| R_HTDHL6 | (3R)-3-Hydroxybutanoly[acyl-carrier protein] hydro lyase | 4.2.1.58 |
| R_HTDR6 | (3R)-3-Hydroxybutanoly[acyl-carrier protein]: NADP + oxidoreductase | 1.1.1.100 |
| R_ILEt6 | ile transport |  |
| R_ILETRS | ile tRNA synthetase | 6.1.1.5. |
| R_IPDDI | Isopentenyl-diphosphate D-isomerase | 5.3.3.2 |
| R_kaasIII | Beta-ketoacyl-ACP synthase III | 2.3.1.180 |
| R_LEUt6 | leu transport |  |
| R_LEUTRS | leu tRNA synthetase | 6.1.1.4 |
| R_LPGS_EFA | lysylphosphatidylglycerol synthetase |  |
| R_LTAS1 | lipoteichoic acid snthase |  |
| R_LTAS2 | lipoteichoic acid modification |  |
| R_LYSTRS | lys tRNA synthetase | 6.1.1.6 |
| R_MACPMT | Malonyl-CoA:[acyl-carrier protein]:S-malonyltransferase | 2.3.1.39 |
| R_MCMAT2 | butyryl[acyl-carrier protein]:malonyl: | 2.3.1.41 |
|  | [acly- carrier protein] C-acetyltransferase |  |
| R_MCMAT3 | hexanoyl[acyl-carrier protein]:malonyl: | 2.3.1.41 |
|  | [acly- carrier protein] C-acetyltransferase |  |
| R_MCMAT4 | octanoyl[acyl-carrier protein]:malonyl: | 2.3.1.41 |
|  | [acly- carrier protein] C-acetyltransferase |  |
| R_MCMAT5 | decanoyl[acyl-carrier protein]:malonyl: | 2.3.1.41 |
|  | [acly- carrier protein] C-acetyltransferase |  |
| R_MCMAT6 | dodecanoyl[acyl-carrier protein]:malonyl: | 2.3.1.41 |
|  | [acly- carrier protein] C-acetyltransferase |  |
| R_MCMAT7 | tetradecanoyl[acyl-carrier protein]:malonyl: | 2.3.1.41 |
|  | [acly- carrier protein] C-acetyltransferase |  |
| R_MCMAT8 | hexadodecanoyl[acyl-carrier protein]:malonyl: | 2.3.1.41 |
|  | [acly- carrier protein] C-acetyltransferase |  |
| R_METTRS | met tRNA synthetase | 6.1.1.10 |
| R_MEVK | mevalonate kinase | 2.7.1.36 |
| R_NACUP | nicotinic acid uptake |  |
| R_NDPK1 | Nucleoside-diphosphate kinase (ATP:GDP) | 2.7.4.6 |
| R_NDPK2 | Nucleoside-diphosphate kinase (ATP:UDP) | 2.7.4.6 |
| R_NDPK3 | Nucleoside-diphosphate kinase (ATP:CDP) | 2.7.4.6 |
| R_NDPK4 | Nucleoside-diphosphate kinase (ATP:dTDP) | 2.7.4.6 |
| R_OCDMAT8 | octadecanonyl[acyl-carrier protein]:malonyl-CoA C-acyltransferase | 1.3.1.10 |
| R_OCMAT3 | octanoyl[acyl-carrier protein]:malonyl-CoA C-acyltransferase | 1.3.1.10 |
| R_PAPPT1_A | Phospho-N-acetylmuramoyl-pentapeptide-transferase | 2.7.8.13 |
| R_PAPPT1_L | Phospho-N-acetylmuramoyl-pentapeptide-transferase | 2.7.8.13 |
| R_PGAMT | phosphoglucosamine mutase | 5.4.2.10 |
| R_PGMT | phosphoglucomutase | 5.4.2.2 |
| R_PGPP_LPL | phosphatidylglycerolphosphate phosphatase | 3.1.3.27 |
| R_PGSA_LPL | phosphatidylglycerol synthase | 2.7.8.5 |
| R_PGSYNTH | peptidoglycan glycosyltransferase |  |
| R_PHETRS | phe tRNA synthetase | 6.1.1.20 |
| R_PMEVK | phosphomevalonat kinase | 2.7.4.2 |
| R_PNTK | pantothenate kinase | 2.7.1.33 |
| R_PPCDC | phosphopantothenoylcysteine decarboxylase | 4.1.1.36 |
| R_PPNCL | Phosphopantothenate-cyteine ligase | 6.3.2.5 |
| R_PROTRS | pro tRNA synthetase | 6.1.1.15 |
| R_PROTS_LPL_v6_0 | protein synthesis |  |
| R_PRPPS | phosphoribosylpyrophosphate synthetase | 2.7.6.1 |
| R_PTPAT | Panthetheine-phosphate adenylyltransferase | 2.7.7.3 |
| R_PYDAMt | pyridoxamine transport |  |
| R_PYDXK | pyridoxal kinase | 2.7.1.35 |
| R_PZS | precursor Z synthase | 4.1.99.18 |
| R_RBT5PDHy | ribitol-5-phosphate 2-dehydrogenase | 1.1.1.137 |
| R_RNAS_LPL | RNA synthesis |  |
| R_SERTRS | ser tRNA synthetase | 6.1.1.11 |
| R_TAPGL4_EFA | wall teichoic acid synthesis |  |
| R_TDMAT6 | tetradecanoyl [acyl-carrier protein]: malonylCoA C-acetyltransferase | 1.3.1.10 |
| R_TDPDRE | dTDP-4-dehydromannose-3,4-epimerase | 5.1.3.13 |
| R_TDPGDH | D-TDP-glucose-4,6-dehydratase | 4.2.1.46 |
| R_THMabc | thm transport via abc system |  |
| R_THRTRS | thr tRNA synthetase | 6.1.1.3 |
| R_TMDS | thymidilate synthase | 2.1.1.45 |
| R_TRDR | thioredoxin reductase | 1.8.1.9 |
| R_TRPTRS | trp tRNA synthetase | 6.1.1.2 |
| R_TYRTRS | tyr tRNA synthetase | 6.1.1.1 |
| R_UAAGLS1 | UDP-N-acetylmuramoyl-L-alanyl-D-glutamate-lysine synthetase | 6.3.2.7 |
| R_UACGE | UDP-N-acetylglucosamine-4-epimerase | 5.1.3.7 |
| R_UAGCVT | UDP-N-acetylglucosamine-1-carboxyvinyltransferase | 2.5.1.7 |
| R_UAGDP | UDP-N-acetylglucosamine diphosphorylase | 2.7.7.23 |
| R_UAGPT1_A | UDP-N-acetylglucosamine-N-acetylmuramyl(pentapeptide) pyrophosphoryl undecaprenol N-acetylglucosamine transferase | 2.4.1.227 |
| R_UAGPT1_L | UDP-N-acetylglucosamine-N-acetylmuramyl(pentapeptide) pyrophosphoryl undecaprenol N-acetylglucosamine transferase | 2.4.1.227 |
| R_UAMAGS | UDP-N-acetylmuramoyl-L-alanyl-glutamate synthetase | 6.3.2.9 |
| R_UAMAS | UDP-N-acetylmuramoyl-L-alanyl-synthetase | 6.3.2.8 |
| R_UAPGR | UDP-N-acetylenolpyrovoylglucosamine reductase | 1.1.1.158 |
| R_UDCPDP | undecaprenyl diphosphatase | 3.6.1.27 |
| R_UDCPDPS | undecaprenyl diphosphate synthase | 2.5.1.31 |
| R_UDPG4E | UDP-glucose 4 epimerase | 5.1.3.2 |
| R_UGLDDS1_A | UDP-N-acetylmuramoyl-alanyl-glutamyl-lysyl-alanyl-alanine synthetase | 6.3.2.10 |
| R_UGLDDS1_L | UDP-N-acetylmuramoyl-alanyl-glutamyl-lysyl-alanyl-alanine synthetase | 6.3.2.10 |
| R_URIDK1 | uridylate kinase | 2.7.4.22 |
| R_VALt6 | val transport |  |
| R_VALTRS | val tRNA synthetase | 6.1.1.9 |
| R_VANB | D-alanine-(R)-lactate ligase | 6.1.2.1 |
| R_WTASI | wall teichoic acid I |  |
| R_WTASII | wall teichoic acid II |  |

**Table S2** Essential proteins.

| ID | Reaction |
| --- | --- |
| EF0043 | Glutamyl-tRNA synthetase |
| EF0045 | Cysteinyl-tRNA synthetase (Cysteine–tRNA ligase) (CysRS) |
| EF0059 | glucosamine-1-phosphate-N-acetyltransferase |
| EF0090 | Diacylglycerol kinase (LPL specific) |
| EF0168 | pantothenate kinase |
| EF0268 | Lysyl-tRNA synthetase |
| EF0680 | peptidoglycan glycosyltransferase |
| EF0724 | glutamyl-tRNA(Gln):L-glutamine amido-ligase (ADP-forming) |
| EF0725 | glutamyl-tRNA(Gln):L-glutamine amido-ligase (ADP-forming) |
| EF0726 | glutamyl-tRNA(Gln):L-glutamine amido-ligase (ADP-forming) |
| EF0727 | Diacylglycerol kinase (LPL specific) |
| EF0739 | Nicotinic acid uptake |
| EF0770 | Phosphatidylglycerol phosphate phosphatase (LPL specific) |
| EF0801 | Leucyl-tRNA synthetase |
| EF0843 | D-alanine-D-alanine ligase (reversible) (D-Ala-D-Ala ligase) |
| EF0845 | UDP-N-acetylmuramoyl-L-alanyl-D-glutamyl-L-lysyl-D-alanyl-D-alanine synthetase |
| EF0849 | alanine racemase |
| EF0880 | dephospho-CoA kinase |
| EF0901 | isopentenyl-diphosphate D-isomerase |
| EF0902 | phosphomevalonate kinase |
| EF0903 | diphosphomevalonate decarboxylase |
| EF0904 | mevalonate kinase |
| EF0930 | Methionyl-tRNA synthetase |
| EF0981 | geranyltranstransferase |
| EF0991 | peptidoglycan glycosyltransferase |
| EF0992 | phospho-N-acetylmuramoyl-pentapeptide-transferase (alpha-glutamate) (D-ala) |
| EF0993 | UDP-N-acetylmuramoyl-L-alanyl-D-glutamate synthetase |
| EF0994 | UDP-N-acetylglucosamine-N-acetylmuramyl-pyrophosphoryl-undecaprenol |
|  | N-acetylglucosamine transferase |
| EF1003 | Isoleucyl-tRNA synthetase |
| EF1036 | nucleoside-diphosphate kinase (ATP:GDP) |
| EF1115 | Phenylalanyl-tRNA synthetase (Phenylalanyl-tRNA synthetase alpha subunit) |
| EF1116 | Phenylalanyl-tRNA synthetase (Phenylalanyl-tRNA synthetase beta subunit) |
| EF1121 | glutamate racemase |
| EF1148 | peptidoglycan glycosyltransferase |
| EF1364 | Hydroxymethylglutaryl CoA reductase |
| EF1379 | Alanyl-tRNA synthetase (AlaRS) |
| EF1391 | precursor Z synthase |
| EF1392 | precursor Z synthase |
| EF1393 | precursor Z synthase |
| EF1396 | precursor Z synthase |
| EF1547 | cytidylate kinase (dCMP) |
| EF1576 | thymidylate synthase |
| EF1577 | dihydrofolate reductase |
| EF1711 | carbonate dehydratase (HCO3 equilibration reaction) |
| EF1746 | UTP-glucose-1-phosphate uridylyltransferase |
| EF1908 | UDP-N-acetylmuramoyl-L-alanine synthetase |
| EF1970 | Aspartyl-tRNA synthetase (AspRS) |
| EF1971 | Histidyl-tRNA synthetase |
| EF2150 | UDP-N-acetylglucosamine 4-epimerase |
| EF2162 | dimethylallyltranstransferase |
| EF2182 | Lipoteichoic acid synthase (LPL specific) |
| EF2183 | Lipoteichoic acid modification (koijbiose) |
| EF2192 | dTDPglucose 4 |
| EF2193 | dTDP-4-dehydromannose 3 |
| EF2194 | glucose-1-phosphate thymidylyltransferase |
| EF2294 | D-alanine-(R)-lactate ligase |
| EF2379 | Prolyl-tRNA synthetase |
| EF2396 | uridylate kinase (UMP) |
| EF2406 | Glycyl-tRNA synthetase |
| EF2407 | Glycyl-tRNA synthetase |
| EF2411 | Diacylglycerol kinase (LPL specific) |
| EF2439 | Undecaprenyl-diphosphatase |
| EF2451 | pantetheine-phosphate adenylyltransferase |
| EF2471 | Arginyl-tRNA synthetase (ArgRS) |
| EF2476 | peptidoglycan glycosyltransferase |
| EF2494 | CDP-Diacylglycerol synthetase (Phosphatidate cytidylyltransferase) (LPL specific) |
| EF2495 | Undecaprenyl diphosphate synthase |
| EF2644 | Diacylglycerol kinase (LPL specific) |
| EF2655 | phosphopantothenate-cysteine ligase |
| EF2656 | phosphopantothenoylcysteine decarboxylase |
| EF2658 | UDP-N-acetylmuramoylpentapeptide-lysine-N6-alanyltransferase |
| EF2661 | Diacylglycerol kinase (LPL specific) |
| EF2691 | 1-Acyl-glycerol-3-phosphate acyltransferase (Efa specific) |
| EF2746 | D-Alanine lipoteichoic acid ligase |
| EF2747 | D-Alanine lipoteichoic acid ligase |
| EF2748 | D-Alanine lipoteichoic acid ligase |
| EF2749 | D-Alanine lipoteichoic acid ligase |
| EF2764 | Thymidylate kinase (dTMP kinase) |
| EF2858 | Threonyl-tRNA synthetase |
| EF2871 | nicotinate-nucleotide adenylyltransferase (nicotinate/ nicotinamide) |
| EF2875 | acetyl-CoA carboxylase |
| EF2876 | acetyl-CoA carboxylase |
| EF2877 | acetyl-CoA carboxylase |
| EF2879 | acetyl-CoA carboxylase |
| EF2882 | Malonyl-CoA:[acyl-carrier-protein] S-malonyltransferase |
| EF2883 | Tetradecanoyl-[acyl-carrier protein]:malonyl-CoA C-acyltransferase |
| EF2885 | beta-ketoacyl-ACP synthase III |
| EF2890 | 2-diacylglycerol 3-glucosyltransferase (LPL specific) |
| EF2891 | 2-diacylglycerol 3-glucosyltransferase (LPL specific) |
| EF2908 | peptidoglycan glycosyltransferase |
| EF2928 | dihydrofolate synthase |
| EF2931 | Valyl-tRNA synthetase |
| EF2973 | alkaline phosphatase |
| EF3072 | Biotin uptake |
| EF3112 | glycerol 3-phosphate acyltransferase (Efa specific) |
| EF3129 | peptidoglycan glycosyltransferase |
| EF3148 | lysylphosphatidyl-glycerol synthetase |
| EF3265 | dihydropteroate synthase |
| EF3267 | GTP cyclohydrolase I |
| EF3268 | 2-amino-4-hydroxy-6-hydroxymethyldihydropteridine diphosphokinase |
| EF3269 | dihydroneopterin aldolase |

**Table S3**: Essential proteins not detected by SWATH-MS. Reactions specific for *E. faecalis* are marked with _EFA.

| ID | Reaction ID | Reaction Name | E.C. # |
| --- | --- | --- | --- |
| EF0168 | R_PNTK | pantothenate kinase | 2.7.1.33 |
| EF0739 | R_NACUP | nicotinic acid uptake, |  |
| EF0904 | R_MEVK | mevalonate kinase | 2.7.1.36 |
| EF0992 | R_PAPPT1_A, | phospho-N-acetylmuramoyl- | 2.7.8.13 |
|  | R_PAPPT1_L | pentapeptide-transferase (L-lysine) | 2.7.8.13 |
| EF1391 | R_PZS | precursor Z synthase |  |
| EF1392 | R_PZS | precursor Z synthase |  |
| EF1393 | R_PZS | precursor Z synthase |  |
| EF1396 | R_PZS | precursor Z synthase, |  |
| EF2162 | R_DMATT | dimethylallyltranstransferase | 2.5.1.1 |
| EF2183 | R_LTAS2 | Lipoteichoic acid modification (koijbiose) |  |
| EF2294 | R_VANB | D-alanine-(R)-lactate ligase | 6.1.2.1 |
| EF2411 | R_DAGK_LPL | diacylglycerol kinase (LPL specific) | 2.7.1.107 |
| EF2439 | R_UDCPK, | undecaprenol kinase, | 2.7.1.66 |
|  | R_UDCPDP | undecaprenyl-diphosphatase | 3.6.1.27 |
| EF2494 | R_DASYN_LPL | CDP-Diacylglycerol synthetase (LPL specific) | 2.7.7.41 |
| EF2661 | R_DAGK_LPL | Diacylglycerol kinase (LPL specific) | 2.7.1.107 |
| EF2748 | R_DARTAL_EFA, | wall teichoic acid modification, |  |
|  | R_DALTAL_EFA | D-alanine lipoteichoic acid ligase | 6.3.2.16 |
| EF2908 | R_PGSYNTH | peptidoglycan glycosyltransferase |  |
| EF3072 | R_BTNt2i | biotin uptake |  |

**Table S4** Non-essential proteins not detected by SWATH-MS.

| ID | Reaction | | |
| --- | --- | --- | --- |
| EF0028 | R_GLCpts, R_MALTpts | |  |
| EF0029 | R_CYSTH, R_CYSTL | |  |
| EF0069 | R_AMANAPE | |  |
| EF0094 | R_FORt, R_NO2t | |  |
| EF0108 | R_ARGORNt | |  |
| EF0273 | R_PGM | |  |
| EF0289 | R_CYSS | |  |
| EF0290 | R_CYSTH, R_CYSTL | |  |
| EF0292 | R_CELBpts | |  |
| EF0387 | R_ASPt6, R_GLUt6 | |  |
| EF0406 | R_MNLpts | |  |
| EF0408 | R_MNLpts | |  |
| EF0411 | R_MNLpts | |  |
| EF0413 | R_M1PD | |  |
| EF0423 | R_HOGA, R_KHGA | |  |
| EF0424 | R_DDGLK | |  |
| EF0433 | R_RMK | |  |
| EF0434 | R_RMI | |  |
| EF0435 | R_RMPA | |  |
| EF0446 | R_MENE | |  |
| EF0448 | R_MEND | |  |
| EF0449 | R_MENH | |  |
| EF0455 | R_MANpts, R_FRUpts | |  |
| EF0456 | R_MANpts, R_FRUpts | |  |
| EF0540 | R_AMANAPE | |  |
| EF0547 | R_NH4t | |  |
| EF0552 | R_MANpts, R_FRUpts | |  |
| EF0553 | R_MANpts, R_FRUpts | |  |
| EF0556 | R_XYLA | |  |
| EF0557 | R_XYLB, R_XYLI | |  |
| EF0576 | R_MNabc | |  |
| EF0628 | R_ACGALpts | |  |
| EF0636 | R_TYRt6 | |  |
| EF0641 | R_LDH_L | |  |
| EF0674 | R_GLYBabc, R_CHLabc | |  |
| EF0675 | R_GLYBabc, R_CHLabc | |  |
| EF0693 | R_F1PK | |  |
| EF0694 | R_FRUpts | |  |
| EF0696 | R_TAGA | |  |
| EF0735 | R_CK | |  |
| EF0806 | R_GLNabc | |  |
| EF0813 | R_GALSZ | |  |
| EF0815 | R_MANpts | |  |
| EF0816 | R_MANpts | |  |
| EF0817 | R_MANpts | |  |
| EF0834 | R_CELBpts | |  |
| EF0837 | R_DHORTS | |  |
| EF0840 | R_DDGLK | |  |
| EF0862 | R_GLYBabc, R_CHLabc | |  |
| EF0921 | R_SO4t4 | |  |
| EF0928 | R_GLCt | |  |
| EF0929 | R_LYSt6 | |  |
| EF0940 | R_FOLt | |  |
| EF0961 | R_P5CRr | |  |
| EF1012 | R_CELBpts | |  |
| EF1013 | R_CELBpts | |  |
| EF1037 | R_ASP2DC | |  |
| EF1053 | R_NO3abc | |  |
| EF1054 | R_NO3abc | |  |
| EF1071 | R_GALTG | |  |
| EF1103 | R_DALAt, R_PROt6, R_DSERt | |  |
| EF1117 | R_GLNabc | |  |
| EF1158 | R_ASNN | |  |
| EF1160 | R_CELBpts | |  |
| EF1173 | R_UNAGAMAMT | |  |
| EF1175 | R_G3PCT | |  |
| EF1207 | R_CITt6 | |  |
| EF1218 | R_SPMDabc, R_PTRCabc | |  |
| EF1219 | R_SPMDabc, R_PTRCabc | |  |
| EF1238 | R_PGDH | |  |
| EF1239 | R_CELBP | |  |
| EF1343 | R_MALTabc | |  |
| EF1344 | R_MALTabc | |  |
| EF1358 | R_GLYCDH, R_ALCD19 | |  |
| EF1386 | R_FORt, R_NO2t | |  |
| EF1388 | R_FDHA | |  |
| EF1389 | R_FDHA | |  |
| EF1390 | R_FDHA | |  |
| EF1395 | R_Moabc | |  |
| EF1398 | R_Moabc | |  |
| EF1399 | R_Moabc | |  |
| EF1541 | R_RIBFLVt2 |  | |
| EF1597 | R_CAT | |  |
| EF1601 | R_SUCpts | |  |
| EF1602 | R_SUCH | |  |
| EF1603 | R_FFSD, R_RAFDH | |  |
| EF1624 | R_ACALD | |  |
| EF1629 | R_EUTB | |  |
| EF1635 | R_ALCD2x | |  |
| EF1662 | R_BUK | |  |
| EF1663 | R_PTB | |  |
| EF1757 | R_PIabc, R_PIhabc | |  |
| EF1758 | R_PIabc, R_PIhabc | |  |
| EF1773 | R_NPPDO | |  |
| EF1779 | R_GARFT | |  |
| EF1802 | R_MANpts | |  |
| EF1803 | R_MANpts | |  |
| EF1828 | R_GLYCt1 | |  |
| EF1834 | R_TAGI | |  |
| EF1835 | R_TAGI | |  |
| EF1836 | R_GALTpts | |  |
| EF1838 | R_GALTpts | |  |
| EF1851 | R_GALSZ | |  |
| EF1904 | R_GPDDA5, R_GPDDA3, R_GPDDA2, R_GPDDA1, R_GPDDA4 | |  |
| EF1920 | R_MALt, R_FUMt | |  |
| EF1952 | R_MANpts | |  |
| EF1953 | R_MANpts | |  |
| EF1969 | R_HMPK1, R_PMPK, R_PYDAMK | |  |
| EF2074 | R_MNabc | |  |
| EF2075 | R_MNabc | |  |
| EF2081 | R_METabc | |  |
| EF2082 | R_METabc | |  |
| EF2163 | R_GPDDA5, R_GPDDA3, R_GPDDA2, R_GPDDA1, R_GPDDA4 | |  |
| EF2198 | R_ACGAMT | |  |
| EF2232 | R_GLYC3Pabc | |  |
| EF2233 | R_GLYC3Pabc | |  |
| EF2257 | R_CELBpts | |  |
| EF2265 | R_PTK, R_PFK | |  |
| EF2266 | R_HOGA, R_KHGA | |  |
| EF2267 | R_ACGALpts, R_ACGApts | |  |
| EF2269 | R_ACGALpts, R_ACGApts | |  |
| EF2270 | R_ACGALpts, R_ACGApts | |  |
| EF2271 | R_ACGALpts, R_ACGApts | |  |
| EF2293 | R_VANX | |  |
| EF2295 | R_LDH_D | |  |
| EF2340 | R_HCYSMT | |  |
| EF2364 | R_XANt2, R_URAt2 | |  |
| EF2377 | R_PROt6 | |  |
| EF2430 | R_XANt2, R_URAt2 | |  |
| EF2442 | R_PIt6, R_PIht6 | |  |
| EF2493 | R_CGPT_EFA | |  |
| EF2497 | R_METDabc | |  |
| EF2498 | R_METDabc | |  |
| EF2560 | R_GLUSYN1 | |  |
| EF2561 | R_DHORD6, R_DHORD | |  |
| EF2566 | R_FRDx | |  |
| EF2573 | R_XANt2, R_URAt2 | |  |
| EF2575 | R_CK | |  |
| EF2581 | R_NADH4 | |  |
| EF2614 | R_ATPS3r | |  |
| EF2641 | R_PROabc, R_GLYBabc | |  |
| EF2642 | R_PROabc, R_GLYBabc | |  |
| EF2646 | R_GLYCK | |  |
| EF2647 | R_GLCNt2 | |  |
| EF2650 | R_SPMDabc, R_PTRCabc | |  |
| EF2651 | R_SPMDabc, R_PTRCabc | |  |
| EF2657 | R_PNTOt2 | |  |
| EF2709 | R_GALSZ | |  |
| EF2721 | R_SERD_L, R_THRD_L | |  |
| EF2767 | R_THMASE | |  |
| EF2773 | R_ARABte | |  |
| EF2775 | R_HMPK1, R_PMPK | |  |
| EF2776 | R_TMPPP | |  |
| EF2777 | R_HETZK | |  |
| EF2905 | R_GLYC3Pabc, R_GLYC3Pabc | |  |
| EF2935 | R_GUAt2, R_HXANt2 | |  |
| EF2959 | R_RIBt2 | |  |
| EF2977 | R_MANpts, R_FRUpts | |  |
| EF2978 | R_MANpts, R_FRUpts | |  |
| EF2980 | R_MANpts, R_FRUpts | |  |
| EF2989 | R_NOX2, R_NOX1 | |  |
| EF3004 | R_SO4t4 | |  |
| EF3015 | R_SERt6 | |  |
| EF3022 | R_GLUt6 | |  |
| EF3029 | R_ACGALpts, R_MANpts, R_ACGApts | |  |
| EF3030 | R_ACGALpts, R_MANpts, R_ACGApts | |  |
| EF3033 | R_ACGALpts, R_MANpts, R_ACGApts | |  |
| EF3042 | R_ACGALpts, R_MANpts, R_ACGApts | |  |
| EF3043 | R_ACGALpts, R_MANpts, R_ACGApts | |  |
| EF3044 | R_AGDC | |  |
| EF3045 | R_ACGALpts, R_MANpts, R_ACGApts | |  |
| EF3046 | R_ACGALpts, R_ACGApts | |  |
| EF3069 | R_FORt | |  |
| EF3090 | R_NNAMr | |  |
| EF3096 | R_NO2t | |  |
| EF3117 | R_TMDPK | |  |
| EF3139 | R_MANpts | |  |
| EF3140 | R_ALCD2x | |  |
| EF3141 | R_PGCD | |  |
| EF3142 | R_PGDH | |  |
| EF3199 | R_METabc | |  |
| EF3200 | R_METabc | |  |
| EF3210 | R_MANpts | |  |
| EF3211 | R_MANpts | |  |
| EF3212 | R_MANpts | |  |
| EF3213 | R_MANpts | |  |
| EF3277 | R_CSNt6 | |  |
| EF3284 | R_CYSTGL | |  |
| EF3285 | R_CELBpts | |  |
| EF3304 | R_F6PA, R_TAL | |  |
| EF3306 | R_SBTpts | |  |
| EF3307 | R_SBTpts | |  |
| EF3310 | R_SBTPD | |  |
| EF3316 | R_ME1x, R_OAADC | |  |

**Table S5** Experimentally determined flux bounds for pH 7.5. Flux bounds are given in
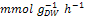
. (*) Reactions, where the model did not allow for the flux bounds as defined in Supplementary Methods 1.15.

| Reaction | Measured uptake / production | Lower Bound | Upper Bound |
| --- | --- | --- | --- |
| R_Ex_glc__e | -5.804 | -6.9648 | -4.6432 |
| R_ex_etoh__e | 2.37 | 1.896 | 2.844 |
| R_Ex_ac__e | 5.566 | 4.4528 | 6.6792 |
| R_Ex_lac_L__e | 0.896 | 0.7168 | 1.0752 |
| R_Ex_for__e | 8.813 | 7.0504 | 10.5756 |
| R_Ex_pyr__e | 0.05 | 0.0249* | 0.05 |
| R_Ex_actn_R__e | - | 0.0027 | 0.0041 |
| R_Ex_asp__e | -0.081 | -0.0972 | -0.0648 |
| R_Ex_ser_e | -0.229 | -0.2748 | -0.1832 |
| R_Ex_glu_L__e | -0.053 | -0.0636 | -0.0424 |
| R_Ex_gly__e | -0.092 | -0.1104 | -0.0736 |
| R_Ex_his_L__e | -0.013 | -0.014 | -0.005* |
| R_Ex_nh4__e | 0.106 | 0.0848 | 0.1272 |
| R_Ex_arg_L__e | -0.206 | -0.2472 | -0.1648 |
| R_Ex_thr_L__e | -0.061 | -0.0732 | -0.0488 |
| R_Ex_ala_L__e | 0.033 | 0.0264 | 0.0396 |
| R_Ex_pro_L__e | -0.052 | -0.052 | -0.006* |
| R_Ex_cys_L__e | 0.032 | 0.01* | 0.0384 |
| R_Ex_tyr_L__e | -0.033 | -0.0396 | -0.0264 |
| R_Ex_val_L__e | -0.083 | -0.0996 | -0.0664 |
| R_Ex_met_L__e | -0.02 | -0.024 | -0.016 |
| R_Ex_orn_L__e | 0.143 | 0.1144 | 0.1716 |
| R_Ex_lys_L__e | -0.082 | -0.0984 | -0.0656 |
| R_Ex_ile_L__e | -0.075 | -0.09 | -0.06 |
| R_Ex_leu_L__e | -0.126 | -0.1512 | -0.1008 |
| R_Ex_phe_L__e | -0.043 | -0.0516 | -0.0344 |
| R_Ex_asn_L__e | -0.011 | -0.0132 | -0.0088 |
| R_Ex_gln_L__e | -0.091 | -0.1092 | -0.0728 |
| R_Ex_trp_L__e | -0.007 | -0.0084 | -0.0056 |
| R_Ex_citr_L__e | 0.007 | 0.0056 | 0.0084 |
|  |  |  |  |
|  |  |  |  |

**Table S6** Experimentally determined flux bounds for pH 6.5. Flux bounds are given in
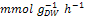
. (*) Reactions, where the model did not allow for the flux bounds as defined in Supplementary Methods 1.15.

| Reaction | Measured uptake / production | Lower Bound | Upper Bound |
| --- | --- | --- | --- |
| R_Ex_glc__e | -7.609 | -9.1308 | -6.0872 |
| R_Ex_etoh__e | 2.036 | 1.6288 | 2.4432 |
| R_Ex_ac__e | 2.682 | 2.1456 | 3.2184 |
| R_Ex_lac_L__e | 5.985 | 4.22* | 8.59* |
| R_Ex_for__e | 5.329 | 3.22* | 6.3948* |
| R_Ex_pyr__e | 0 | 0.0662* | 0.0907* |
| R_Ex_actn_R__e | - | 0.0288 | 0.0449 |
| R_Ex_asp__e | -0.036 | -0.0432 | -0.0288 |
| R_Ex_ser_e | -0.299 | -0.3588 | -0.2392 |
| R_Ex_glu_L__e | -0.009 | -0.0108 | -0.0072 |
| R_Ex_gly__e | -0.074 | -0.0888 | -0.0592 |
| R_Ex_his_L__e | -0.003 | -0.0036 | -0.0024 |
| R_Ex_nh4__e | 0.284 | 0.2272 | 0.3408 |
| R_Ex_arg_L__e | -0.271 | -0.3252 | -0.2168 |
| R_Ex_thr_L__e | -0.052 | -0.0624 | -0.0416 |
| R_Ex_ala_L__e | 0.086 | 0.0688 | 0.1032 |
| R_Ex_pro_L__e | -0.039 | -0.0468 | -0.0312 |
| R_Ex_cys_L__e* | 0.048 | 0.01* | 0.56 |
| R_Ex_tyr_L__e | -0.037 | -0.0444 | -0.0296 |
| R_Ex_val_L__e | -0.065 | -0.078 | -0.052 |
| R_Ex_met_L__e | -0.022 | -0.0264 | -0.0176 |
| R_Ex_orn_L__e | 0.217 | 0.1736 | 0.2604 |
| R_Ex_lys_L__e | -0.066 | -0.0792 | -0.0528 |
| R_Ex_ile_L__e | -0.07 | -0.084 | -0.056 |
| R_Ex_leu_L__e | -0.125 | -0.15 | -0.1 |
| R_Ex_phe_L__e | -0.054 | -0.0648 | -0.0432 |
| R_Ex_asn_L__e | -0.004 | -0.0048 | -0.0032 |
| R_Ex_gln_L__e | -0.104 | -0.1248 | -0.0832 |
| R_Ex_trp_L__e | -0.001 | -0.003* | -0.0008 |
| R_Ex_citr_L__e | 0.007 | 0.0056 | 0.0084 |

**Table S7** New pH-specific reactions introduced to the genome-scale model of *Enterococcus faecalis* V583. The directionality of reversible reactions is determined by the flux boundaries as listed in Tab S8.

| Reaction ID | Reaction Name | Equation | Reversible |
| --- | --- | --- | --- |
| R_Proton_leak | Proton leak across the membrane | M_h_e 🡪 M_h_c | True |
| R_LACLt_pH75 | L-lactate transport in/out via proton symporter for pH 7.5 | M_lac_L_c + M_h_c 🡪 M_lac_L_e + M_h_e | True |
| R_Ex_pih__e | Phosphate exchange 2 | 🡪 M_pih_e | True |
| R_Piht6 | phosphate transport in/out via proton symporter for pH 6.5 | M_pih_e + M_h_e 🡪 M_pi_c + 2 M_h_c | True |
| R_Pisink | Phosphate sink reaction | M_pi_c 🡪 | True |

**Table S8:** Overview of pH dependent processes with notable changes during the pH shift experiment, which were incorporated into the genome-scale model. Flux bounds (LB: lower bound, UB: upper bound) are given in
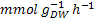
. Newly defined reactions are listed in Tab S7.

|  |  | pH 7.5 |  | pH 6.5 |  |
| --- | --- | --- | --- | --- | --- |
| Reaction ID | Reaction Name | LB | UB | LB | UB |
| R_Proton_leak | Proton leak across the membrane | 0.224 | 1 | 0.612 | 2 |
| R_PIt6 | Hydrogenphosphate transport via proton symporter | 0.8 | 2 | 0 | 0 |
| R_PIht6 | Dihydrogenphosphate transport via proton symporter | 0 | 0 | 0.8 | 2 |
| R_Ex_pih__e | Exchange reaction for dihydrogenphosphate | -10 | 10 | -10 | 10 |
| R_Ex_pi__e | Exchange reaction for hydrogenphosphate | -10 | 10 | -10 | 10 |
| R_PIsink | Phosphate sink reaction | 0.6 | 2 | 0.6 | 2 |
| R_LACLt | L-lactate transport via proton symporter | 0 | 0 | -10 | 10 |
| R_LACLt_pH7.5 | L-lactate transport via proton symporter | -10 | 10 | 0 | 0 |

**Table S9:** Reactivated proteins with the respective associated reaction. Non-membrane-associated proteins are marked with *.

| ID | Reaction ID | Reaction name | E.C. # |
| --- | --- | --- | --- |
| EF0108 | R_ARGORNt | arginine ornithine antiporter |  |
| EF0387 | R_ASPt6 | L-aspartate transport in via proton symport |  |
| EF0547 | R_NH4t | ammonium transporter family protein |  |
| EF0929 | R_LYSt6 | L-lysine transport in/out via proton symport |  |
| EF1103 | R_PROt6 | L-proline transport in/out via proton symport |  |
| EF1386 | R_FORt, | formate transport, |  |
|  | R_NO2t | nitrite transport |  |
| EF2364 | R_XANt2 | hypoxanthine transport in via proton symport |  |
| EF2442 | R_PIt6 | phosphate transport in/out via proton symporter |  |
| EF2614 | R_ATPS3r | ATP synthase V-type (three protons for one ATP) | 3.6.3.14 |
| EF3015 | R_SERt6 | L-serine transport in/out via proton symport |  |
| EF3117* | R_TMDPK | thiamine diphosphokinase | 2.7.6.2 |
| EF3199 | R_METabc | L-methionine transport via ABC system |  |
| EF3200 | R_METabc | L-methionine transport via ABC system |  |
| EF3284* | R_CYSTGL | cystathionine g-lyase | 4.4.1.1 |
| EF3304* | R_F6PA, | fructose 6-phosphate aldolase, |  |
|  | R_TAL | transaldolase | 2.2.1.2 |

**Table S10** Significant protein concentration changes in comparison to t_1_ per time point.

| Time Point | Reaction ID | Protein ID | Concentration Change | p-value |
| --- | --- | --- | --- | --- |
| t_2_ | R_CYSS | EF1584 | 2.6445 | 0.0488 |
|  | R_GRTT | EF0981 | 2.2317 | 0.0488 |
|  | R_ME1x | EF1206 | 1.8574 | 0.0488 |
|  | R_OAADC | EF1206 | 1.8574 | 0.0488 |
| t_3_ | R_ACACT1 | EF1364 | 2.8159 | 0.0122 |
|  | R_ACKr | EF1983 | 2.4542 | 0.0198 |
|  | R_ACTPASE | EF2401 | 0.5749 | 0.0166 |
|  | R_ADPT | EF1687 | 1.7817 | 0.0065 |
|  | R_AGDC | EF1317 | 2.9243 | 0.0305 |
|  | R_AICART | EF1778 | 2.8364 | 0.0123 |
|  | R_ARGD | EF0104 | 1.6692 | 0.0256 |
|  | R_ASNTAL | EF0726 | 3.2725 | 0.0049 |
|  | R_ASPCT | EF2755 | 0.5502 | 0.0125 |
|  | R_BPPA2 | EF2150 | 0.6141 | 0.0126 |
|  | R_BPPA2_L | EF2150 | 0.6141 | 0.0126 |
|  | R_CDD | EF0175 | 0.5444 | 0.0024 |
|  | R_CDPMEK | EF0051 | 2.9391 | 0.0426 |
|  | R_CELBpts | EF1769 | 0.5252 | 0.0049 |
|  | R_CELBpts | EF1769 | 0.5252 | 0.0049 |
|  | R_CITL | EF3320 | 3.6885 | 0.0087 |
|  | R_CITt6 | EF3327 | 0.4815 | 0.0049 |
|  | R_CSND | EF0390 | 2.4557 | 0.0131 |
|  | R_CYSS | EF1584 | 3.1364 | 0.0049 |
|  | R_DEX_PS_EFA | EF2150 | 0.6141 | 0.0126 |
|  | R_DPMVD | EF0903 | 3.44 | 0.0166 |
|  | R_EPA_PS_EFA | EF2150 | 0.6141 | 0.0126 |
|  | R_FTHFL | EF1725 | 4.719 | 0.0065 |
|  | R_G1PACT | EF0059 | 2.1758 | 0.0319 |
|  | R_G6PI | EF1416 | 2.2344 | 0.0361 |
|  | R_GALTG | EF2782 | 2.4589 | 0.027 |
|  | R_GALU | EF1746 | 1.8505 | 0.0259 |
|  | R_GAT1_EFA | EF3112 | 1.8775 | 0.0049 |
|  | R_GHMT | EF2550 | 2.6084 | 0.0168 |
|  | R_GLNTAL | EF0726 | 3.2725 | 0.0049 |
|  | R_GRTT | EF0981 | 2.6644 | 0.0049 |
|  | R_HMGCOAR | EF1364 | 2.8159 | 0.0122 |
|  | R_IMPC | EF1778 | 2.8364 | 0.0123 |
|  | R_LDH_L | EF0255 | 3.1282 | 0.0082 |
|  | R_MALTabc | EF1345 | 2.5311 | 0.016 |
|  | R_MAN6PI | EF2589 | 2.1032 | 0.038 |
|  | R_ME1x | EF1206 | 2.252 | 0.0024 |
|  | R_METTRS | EF0930 | 2.5864 | 0.0285 |
|  | R_MNOH | EF3135 | 2.484 | 0.0499 |
|  | R_OAADC | EF1206 | 2.252 | 0.0024 |
|  | R_OCBT | EF2755 | 0.5502 | 0.0125 |
|  | R_PANB | EF1860 | 1.8729 | 0.0054 |
|  | R_PDH | EF1355 | 5.2722 | 0.0035 |
|  | R_PFL | EF1613 | 2.2651 | 0.0098 |
|  | R_PGI | EF1416 | 2.2344 | 0.0361 |
|  | R_PGL | EF1918 | 0.5949 | 0.0087 |
|  | R_PGPP_LPL | EF0770 | 0.5951 | 0.0411 |
|  | R_PPM2 | EF0185 | 2.3616 | 0.0049 |
|  | R_PYK | EF1046 | 2.8141 | 0.0374 |
|  | R_SDPDS | EF3178 | 3.3402 | 0.038 |
|  | R_SHK3D | EF1561 | 0.5857 | 0.0375 |
|  | R_TAGA | EF1807 | 1.9337 | 0.0256 |
|  | R_TAPGL4_EFA | EF2755 | 0.5502 | 0.0125 |
|  | R_UACGE | EF2150 | 0.6141 | 0.0126 |
|  | R_UAGDP | EF0059 | 2.1758 | 0.0319 |
|  | R_UDPG4E | EF1070 | 2.3004 | 0.0087 |
|  | R_VALTRS | EF2931 | 2.6038 | 0.0194 |
| t_4_ | R_ACACT1 | EF1364 | 2.4426 | 0.0237 |
|  | R_ACKr | EF1983 | 2.2097 | 0.0316 |
|  | R_ACTPASE | EF2401 | 0.562 | 0.0139 |
|  | R_ADPT | EF1687 | 1.7044 | 0.0086 |
|  | R_ADSL1 | EF2361 | 2.4672 | 0.0473 |
|  | R_ADSL2 | EF2361 | 2.4672 | 0.0473 |
|  | R_AGDC | EF1317 | 2.5506 | 0.0498 |
|  | R_AHCYSNS | EF2694 | 0.4252 | 0.0302 |
|  | R_AICART | EF1778 | 2.4735 | 0.0237 |
|  | R_ARGD | EF0104 | 1.7834 | 0.014 |
|  | R_ASNTAL | EF0726 | 3.0329 | 0.0064 |
|  | R_BPPA2 | EF2150 | 0.6377 | 0.0182 |
|  | R_BPPA2_L | EF2150 | 0.6377 | 0.0182 |
|  | R_CDD | EF0175 | 0.5676 | 0.0037 |
|  | R_CELBpts | EF1769 | 0.4556 | 0.0021 |
|  | R_CELBpts | EF1769 | 0.4556 | 0.0021 |
|  | R_CITL | EF3320 | 3.6568 | 0.0086 |
|  | R_CSND | EF0390 | 2.081 | 0.0316 |
|  | R_CYSS | EF1584 | 3.0174 | 0.0064 |
|  | R_DEX_PS_EFA | EF2150 | 0.6377 | 0.0182 |
|  | R_DHNPA | EF3269 | 0.3038 | 0.0076 |
|  | R_DPMVD | EF0903 | 3.3698 | 0.0173 |
|  | R_EPA_PS_EFA | EF2150 | 0.6377 | 0.0182 |
|  | R_FMETDF | EF3066 | 0.5906 | 0.0173 |
|  | R_FTHFL | EF1725 | 3.6401 | 0.014 |
|  | R_G1PACT | EF0059 | 2.1913 | 0.0302 |
|  | R_G6PI | EF1416 | 2.2958 | 0.0302 |
|  | R_GALTG | EF2782 | 2.1283 | 0.0498 |
|  | R_GAT1_EFA | EF3112 | 1.7018 | 0.0099 |
|  | R_GHMT | EF2550 | 2.4204 | 0.0247 |
|  | R_GLNS | EF2159 | 0.6516 | 0.0365 |
|  | R_GLNTAL | EF0726 | 3.0329 | 0.0064 |
|  | R_GRTT | EF0981 | 2.0718 | 0.0165 |
|  | R_HMGCOAR | EF1364 | 2.4426 | 0.0237 |
|  | R_IMPC | EF1778 | 2.4735 | 0.0237 |
|  | R_LDH_L | EF0255 | 3.2884 | 0.0076 |
|  | R_MALTabc | EF1345 | 2.503 | 0.0166 |
|  | R_MAN6PI | EF2589 | 2.0617 | 0.0407 |
|  | R_ME1x | EF1206 | 2.3248 | 0.0021 |
|  | R_MTAN | EF2694 | 0.4252 | 0.0302 |
|  | R_OAADC | EF1206 | 2.3248 | 0.0021 |
|  | R_PANB | EF1860 | 1.8698 | 0.0064 |
|  | R_PDH | EF1355 | 5.7175 | 0.0027 |
|  | R_PFL | EF1613 | 2.8061 | 0.0054 |
|  | R_PGDH | EF1243 | 1.8384 | 0.0076 |
|  | R_PGI | EF1416 | 2.2958 | 0.0302 |
|  | R_PGL | EF1918 | 0.5602 | 0.0064 |
|  | R_PGPP_LPL | EF0770 | 0.4609 | 0.0076 |
|  | R_PPM2 | EF0185 | 2.119 | 0.0086 |
|  | R_TAGA | EF1807 | 1.7662 | 0.0407 |
|  | R_UACGE | EF2150 | 0.6377 | 0.0182 |
|  | R_UAGDP | EF0059 | 2.1913 | 0.0302 |
|  | R_VALTRS | EF2931 | 3.01 | 0.0095 |
| t_5_ | R_ACACT1 | EF1364 | 3.0177 | 0.0068 |
|  | R_ACCOAC | EF2879 | 0.5329 | 0.0122 |
|  | R_ACKr | EF1983 | 2.7272 | 0.0088 |
|  | R_ACTPASE | EF2401 | 0.4196 | 0.0016 |
|  | R_ADPT | EF1687 | 1.6879 | 0.0073 |
|  | R_ADSL1 | EF2361 | 2.5939 | 0.0297 |
|  | R_ADSL2 | EF2361 | 2.5939 | 0.0297 |
|  | R_AGDC | EF1317 | 2.8468 | 0.0249 |
|  | R_AHCYSNS | EF2694 | 0.4727 | 0.0375 |
|  | R_AICART | EF1778 | 2.6057 | 0.0137 |
|  | R_ALATRS | EF1379 | 3.1925 | 0.023 |
|  | R_ARGD | EF0104 | 1.7297 | 0.0141 |
|  | R_ARGTRS | EF2471 | 2.3586 | 0.0443 |
|  | R_ASNTAL | EF0726 | 2.6243 | 0.0088 |
|  | R_ASPCT | EF2755 | 0.4149 | 0.0016 |
|  | R_CDD | EF0175 | 0.471 | 0.0003 |
|  | R_CDPMEK | EF0051 | 4.5887 | 0.0075 |
|  | R_CELBpts | EF1769 | 0.4848 | 0.0016 |
|  | R_CELBpts | EF1769 | 0.4848 | 0.0016 |
|  | R_CITL | EF3320 | 3.7044 | 0.0068 |
|  | R_CITt6 | EF3327 | 0.537 | 0.0075 |
|  | R_CSND | EF0390 | 2.489 | 0.0094 |
|  | R_CYSS | EF1584 | 2.7614 | 0.0068 |
|  | R_DAGK_LPL | EF2644 | 2.695 | 0.0118 |
|  | R_DHNPA | EF3269 | 0.4327 | 0.0275 |
|  | R_DPMVD | EF0903 | 3.3498 | 0.0141 |
|  | R_FTHFL | EF1725 | 4.0139 | 0.0075 |
|  | R_G1PACT | EF0059 | 2.0032 | 0.0361 |
|  | R_G6PI | EF1416 | 2.1746 | 0.0302 |
|  | R_GALTG | EF2782 | 2.4466 | 0.0209 |
|  | R_GALU | EF1746 | 1.948 | 0.0137 |
|  | R_GAT1_EFA | EF3112 | 1.7422 | 0.0068 |
|  | R_GHMT | EF2550 | 2.5413 | 0.0149 |
|  | R_GLNS | EF2159 | 0.6526 | 0.0295 |
|  | R_GLNTAL | EF0726 | 2.6243 | 0.0088 |
|  | R_GLYTRS | EF2406 | 3.786 | 0.0128 |
|  | R_GRTT | EF0981 | 2.4334 | 0.0064 |
|  | R_HBUR1 | EF2881 | 1.6221 | 0.0209 |
|  | R_HDDR5 | EF2881 | 1.6221 | 0.0209 |
|  | R_HDER4 | EF2881 | 1.6221 | 0.0209 |
|  | R_HHDR7 | EF2881 | 1.6221 | 0.0209 |
|  | R_HHYR2 | EF2881 | 1.6221 | 0.0209 |
|  | R_HMGCOAR | EF1364 | 3.0177 | 0.0068 |
|  | R_HOCR3 | EF2881 | 1.6221 | 0.0209 |
|  | R_HODR8 | EF2881 | 1.6221 | 0.0209 |
|  | R_HTDR6 | EF2881 | 1.6221 | 0.0209 |
|  | R_ILETRS | EF1003 | 2.8801 | 0.0295 |
|  | R_IMPC | EF1778 | 2.6057 | 0.0137 |
|  | R_LDH_L | EF0255 | 3.181 | 0.0068 |
|  | R_LEUTRS | EF0801 | 2.6186 | 0.0386 |
|  | R_MALTabc | EF1345 | 2.5972 | 0.0108 |
|  | R_MAN6PI | EF2589 | 2.1654 | 0.0249 |
|  | R_MCMAT2 | EF0283 | 3.3468 | 0.0191 |
|  | R_MCMAT3 | EF0283 | 3.3468 | 0.0191 |
|  | R_MCMAT4 | EF0283 | 3.3468 | 0.0191 |
|  | R_MCMAT5 | EF0283 | 3.3468 | 0.0191 |
|  | R_MCMAT6 | EF0283 | 3.3468 | 0.0191 |
|  | R_MCMAT7 | EF0283 | 3.3468 | 0.0191 |
|  | R_MCMAT8 | EF0283 | 3.3468 | 0.0191 |
|  | R_ME1x | EF1206 | 2.0557 | 0.0031 |
|  | R_METTRS | EF0930 | 2.1919 | 0.0444 |
|  | R_MTAN | EF2694 | 0.4727 | 0.0375 |
|  | R_NNAMr | EF3192 | 0.5788 | 0.034 |
|  | R_NPPDO | EF2881 | 1.6221 | 0.0209 |
|  | R_OAADC | EF1206 | 2.0557 | 0.0031 |
|  | R_OCBT | EF2755 | 0.4149 | 0.0016 |
|  | R_ORPT | EF1712 | 1.3968 | 0.0466 |
|  | R_PANB | EF1860 | 1.8901 | 0.0044 |
|  | R_PDH | EF1355 | 6.1474 | 0.0016 |
|  | R_PFL | EF1613 | 2.0358 | 0.0149 |
|  | R_PGDH | EF1243 | 1.9047 | 0.0045 |
|  | R_PGI | EF1416 | 2.1746 | 0.0302 |
|  | R_PGL | EF1918 | 0.6864 | 0.0275 |
|  | R_PGPP_LPL | EF0770 | 0.5387 | 0.0158 |
|  | R_PPM2 | EF0185 | 1.9564 | 0.0117 |
|  | R_PRFGS | EF1784 | 0.2511 | 0.0068 |
|  | R_PTAr | EF0949 | 2.3717 | 0.0249 |
|  | R_PYK | EF1046 | 2.6537 | 0.0344 |
|  | R_RNTR1 | EF2755 | 0.4149 | 0.0016 |
|  | R_RNTR2 | EF2755 | 0.4149 | 0.0016 |
|  | R_RNTR3 | EF2755 | 0.4149 | 0.0016 |
|  | R_RNTR4 | EF2755 | 0.4149 | 0.0016 |
|  | R_SBTpts | EF2603 | 0.4766 | 0.0068 |
|  | R_SDPDS | EF3178 | 3.1882 | 0.0341 |
|  | R_TAGA | EF1807 | 2.0684 | 0.0118 |
|  | R_TAPGL4_EFA | EF2755 | 0.4149 | 0.0016 |
|  | R_TREpts | EF2438 | 0.426 | 0.0166 |
|  | R_UAGDP | EF0059 | 2.0032 | 0.0361 |
|  | R_UDPG4E | EF1070 | 2.3418 | 0.0068 |
|  | R_VALTRS | EF2931 | 3.0715 | 0.0071 |
| t_6_ | R_ACACT1 | EF1364 | 2.5878 | 0.0145 |
|  | R_ACKr | EF1983 | 2.4454 | 0.0159 |
|  | R_ACTPASE | EF2401 | 0.3865 | 0.0007 |
|  | R_ADPT | EF1687 | 1.8624 | 0.0038 |
|  | R_ADSL1 | EF2361 | 2.6182 | 0.0312 |
|  | R_ADSL2 | EF2361 | 2.6182 | 0.0312 |
|  | R_AGDC | EF1317 | 2.7169 | 0.033 |
|  | R_AICART | EF1778 | 2.3469 | 0.0221 |
|  | R_ARGD | EF0104 | 1.78 | 0.0123 |
|  | R_ASNTAL | EF0726 | 2.9769 | 0.0051 |
|  | R_BPPA2 | EF2150 | 0.6777 | 0.0272 |
|  | R_BPPA2_L | EF2150 | 0.6777 | 0.0272 |
|  | R_CDD | EF0175 | 0.4816 | 0.0007 |
|  | R_CELBpts | EF1769 | 0.4289 | 0.0007 |
|  | R_CELBpts | EF1769 | 0.4289 | 0.0007 |
|  | R_CITL | EF3320 | 3.5937 | 0.0081 |
|  | R_CITt6 | EF3321 | 0.5687 | 0.0174 |
|  | R_CSND | EF0390 | 1.9408 | 0.0408 |
|  | R_CTPS2 | EF1147 | 1.6677 | 0.0388 |
|  | R_CYSS | EF1584 | 2.6021 | 0.0092 |
|  | R_DEX_PS_EFA | EF2150 | 0.6777 | 0.0272 |
|  | R_DHNPA | EF3269 | 0.4818 | 0.0494 |
|  | R_DPMVD | EF0903 | 3.1562 | 0.0174 |
|  | R_EPA_PS_EFA | EF2150 | 0.6777 | 0.0272 |
|  | R_FTHFL | EF1725 | 3.8699 | 0.0097 |
|  | R_G1PACT | EF0059 | 2.3901 | 0.0159 |
|  | R_G6PI | EF1416 | 2.3386 | 0.0219 |
|  | R_GALTG | EF2782 | 2.3791 | 0.023 |
|  | R_GAT1_EFA | EF3112 | 1.6195 | 0.0139 |
|  | R_GF6PTA | EF2151 | 1.4299 | 0.0445 |
|  | R_GHMT | EF2550 | 2.3782 | 0.0209 |
|  | R_GLNTAL | EF0726 | 2.9769 | 0.0051 |
|  | R_GLYTRS | EF2406 | 3.328 | 0.0205 |
|  | R_GRTT | EF0981 | 2.6118 | 0.0038 |
|  | R_HMGCOAR | EF1364 | 2.5878 | 0.0145 |
|  | R_IMPC | EF1778 | 2.3469 | 0.0221 |
|  | R_IPDDI | EF0901 | 0.4982 | 0.0388 |
|  | R_LDH_L | EF0255 | 3.0627 | 0.0075 |
|  | R_MAN6PI | EF2589 | 2.3056 | 0.0186 |
|  | R_ME1x | EF1206 | 2.0801 | 0.0028 |
|  | R_METTRS | EF0930 | 2.5395 | 0.0231 |
|  | R_MTHPTGHM | EF0395 | 2.0973 | 0.033 |
|  | R_OAADC | EF1206 | 2.0801 | 0.0028 |
|  | R_PANB | EF1860 | 1.8087 | 0.0054 |
|  | R_PDH | EF1355 | 6.1987 | 0.0009 |
|  | R_PFL | EF1612 | 2.03 | 0.0051 |
|  | R_PGI | EF1416 | 2.3386 | 0.0219 |
|  | R_PGPP_LPL | EF0770 | 0.5288 | 0.0152 |
|  | R_PPM2 | EF0185 | 2.087 | 0.0082 |
|  | R_PRFGS | EF1784 | 0.2469 | 0.006 |
|  | R_PTAr | EF0949 | 2.193 | 0.0399 |
|  | R_PYK | EF1046 | 2.5334 | 0.0467 |
|  | R_SBTpts | EF2603 | 0.4115 | 0.0037 |
|  | R_SDPDS | EF3178 | 3.363 | 0.0312 |
|  | R_TAGA | EF1807 | 1.9763 | 0.0163 |
|  | R_TDPDRR | EF2191 | 1.4179 | 0.0419 |
|  | R_TRDR | EF1338 | 2.8143 | 0.0208 |
|  | R_UACGE | EF2150 | 0.6777 | 0.0272 |
|  | R_UAGDP | EF0059 | 2.3901 | 0.0159 |
|  | R_UDPG4E | EF1070 | 2.2326 | 0.0091 |
|  | R_VALTRS | EF2931 | 2.703 | 0.0134 |
| t_7_ | R_ACKr | EF1983 | 2.4075 | 0.0352 |
|  | R_ACTPASE | EF2401 | 0.4969 | 0.0131 |
|  | R_ADPT | EF1687 | 1.7913 | 0.0131 |
|  | R_ASNTAL | EF0726 | 2.8045 | 0.0166 |
|  | R_CDD | EF0175 | 0.4266 | 0.0001 |
|  | R_CYSS | EF1584 | 2.2728 | 0.0352 |
|  | R_FMNAT | EF1295 | 0.6753 | 0.0374 |
|  | R_FTHFL | EF1725 | 2.9021 | 0.0486 |
|  | R_G6PI | EF1416 | 2.2811 | 0.0486 |
|  | R_GAT1_EFA | EF3112 | 1.7189 | 0.0184 |
|  | R_GLNTAL | EF0726 | 2.8045 | 0.0166 |
|  | R_LDH_L | EF0255 | 2.8932 | 0.02 |
|  | R_ME1x | EF1206 | 1.6292 | 0.0352 |
|  | R_OAADC | EF1206 | 1.6292 | 0.0352 |
|  | R_PANB | EF1860 | 1.6977 | 0.0202 |
|  | R_PDH | EF1355 | 6.1928 | 0.0032 |
|  | R_PFL | EF1612 | 2.3424 | 0.0074 |
|  | R_PGI | EF1416 | 2.2811 | 0.0486 |
|  | R_PPM2 | EF0185 | 2.2648 | 0.0134 |
|  | R_RBFK | EF1295 | 0.6753 | 0.0374 |
|  | R_VALTRS | EF2931 | 2.5382 | 0.0352 |
| t_8_ | R_ACTPASE | EF2401 | 0.4252 | 0.0013 |
|  | R_ADPT | EF1687 | 1.6662 | 0.0149 |
|  | R_ADSS | EF0014 | 0.5674 | 0.0075 |
|  | R_AICART | EF1778 | 0.41 | 0.0421 |
|  | R_ASPK | EF0368 | 0.4923 | 0.0202 |
|  | R_CDD | EF0175 | 0.4768 | 0.0001 |
|  | R_GAT1_EFA | EF3112 | 1.5759 | 0.0379 |
|  | R_GLUDy | EF1415 | 0.609 | 0.0395 |
|  | R_GLYK | EF1929 | 0.5845 | 0.0448 |
|  | R_IMPC | EF1778 | 0.41 | 0.0421 |
|  | R_PDE1 | EF0063 | 0.4704 | 0.0028 |
|  | R_PDE2 | EF0063 | 0.4704 | 0.0028 |
|  | R_PDE3 | EF0063 | 0.4704 | 0.0028 |
|  | R_PDE4 | EF0063 | 0.4704 | 0.0028 |
|  | R_PDE5 | EF0063 | 0.4704 | 0.0028 |
|  | R_PFL | EF1612 | 3.057 | 0.0001 |
|  | R_PGL | EF1918 | 1.7277 | 0.0092 |
|  | R_PPM2 | EF0185 | 2.046 | 0.0156 |
|  | R_PRFGS | EF1784 | 0.1896 | 0.0028 |
|  | R_RHC | EF1182 | 1.7792 | 0.0319 |
|  | R_UDPG4E | EF1070 | 2.3293 | 0.0109 |

# Proteome Integration

In order to integrate proteomic data into genome-scale models, the respective model needs to contain curated gene-protein-reaction associations (GPRs). GPRs link the proteomic data to the genome-scale model by facilitating the mapping process between detected proteins and the respective model reactions. Reactions without an annotated GPR cannot be evaluated using proteomic data and introduce a degree of uncertainty in the model analysis. Thus, the quality and extend of the GPRs annotated in the genome-scale metabolic model largely determine the potential gain in integrating proteomic data. By introducing GPRs to the genome-scale model we can evaluate a reaction’s activity based on the detected proteins in the proteomic data. Using ’AND’ and ’OR’ relationships between individual proteins, we can even assess the presence of multi-enzyme complexes and isoforms.

Furthermore, the model needs to be adapted to the experimental design. A model, where changing environmental conditions have no influence on the model itself, is insufficient to deal with a chemostat shift experiment specifically designed to evaluate an organism’s adaptive process to alternate environmental conditions.

Essential reactions and essential proteins provide a measure to control the quality of the proteomic data

Constraint-based modeling techniques such as flux balance analysis (FBA) or flux variability analysis (FVA) will only result in a feasible model solution, if we prohibit the inactivation of essential reactions and proteins during the mapping process. Thus, a protein and reaction essentiality scan is a necessary prerequisite prior to the integration of proteomic data preventing the automatic inactivation of essential proteins and reactions. However, instead of excluding essential reactions and proteins completely from the integration process, we use the knowledge to assess the quality and extend of the proteomic data.

To assess the quality of the proteomic data we determine the extent of the SWATH-MS library as well as the individual proteomic samples and analyze the essential proteins not detected in the samples. The extent of both the SWATH-MS library and the individual samples indicates the efficiency of the sample preparation process. The SWATH-MS library in particular represents the maximum amount of proteins that can be identified in the proteomic samples. Essential proteins that cannot be found in the mass spectrometry data either suggest an artifact introduced to the model or give some information about the quality of the proteomic data. Model artifacts include potentially missing reactions, which cause normally non-essential reactions with existing parallel pathways to be essential. The comparison of the proteomic data to the list of essential reactions may also be used to assess the quality of the sample preparation.

Before integrating any kind of proteomic data, and as always done in the context of the analysis of whole-genome scale models metabolic constraints are applied, usually in the form of chemostat data. Thereby we can check the model’s solution with a determined growth rate before restricting the amount of allowed reactions. For the case study used here, where we use different sets of experimental data representing different steady states the second set of metabolic constraints is applied only after computing an initial set of flux boundaries for the first experimental condition with the mapped proteome but before applying determined significant fold changes in the protein concentrations.

During the mapping process of the proteomic data any reaction without sufficient protein evidence is inactivated. Exchange reactions and essential proteins are exempt from the data integration as the first have no annotated GPRs and the latter are required for the model to have a feasible solution.

To integrate the first level of the proteomic data, each reaction is called individually and the corresponding GPR string is retrieved. The annotated proteins are first compared to a list of essential proteins. Barring a match, a second comparison is drawn with the detected proteins in the proteomic data. If the particular protein is not listed in the proteomic data, it is inactivated and the corresponding reaction’s activity is evaluated based on the presence of the remaining proteins in the GPR. If the reaction is inactive after all entries in the GPR have been analyzed, the reaction’s flux boundaries are set to zero.

After all proteins not detected in the proteomic data are inactivated, it is likely that the model does not have a feasible solution within the metabolic constraints. The application of metabolic and proteomic constraints creates additional essentialities in the genome-scale model as we limit its ability to compensate inactivated reactions. Therefore, selected proteins have to be reactivated to ensure that the model has a feasible solution and fits the growth parameters of the chemostat culture. In general, this is a manual process specific to each model and each data set.

We established a number of criteria that aid in selecting the proteins to be reactivated:

- In general:
  - Are other proteins of the particular metabolic pathway present?
  - Is the protein of interest a transmembrane protein and or associated to the membrane?
- For proteins, which are part of a multi-protein complex:
  - Are other proteins of the complex present?
- For proteins, which are part of a transporter complex:
  - Is the transported model species measured in the chemostat data?
  - Is the transported model species an essential medium component?

Any of these criteria questions suggests that the protein is most likely present in the organism even though it is not detected during MS analysis. The protein of interest can be reactivated in the model without introducing too much of a bias, particularly as the list of reactivated proteins can also be used assess the efficiency of the sample preparation process and the model’s quality.

Applying protein quantity changes directs the model to represent adaptive processes

At this point the genome-scale metabolic model contains two layers of constraints beyond those imposed by the model itself: the metabolic chemostat data and the mapped proteomic data. In this configuration, an initial set of flux boundaries is calculated via FVA, which serves as the basis for the application of fold changes and represents the model’s solution for the first experimental condition. We then apply the second set of metabolic constraints before integrating the protein quantity changes. The aim of this step is to ensure that proteome changes, which accompany the metabolic adaptation, are represented in the genome-scale model and increase its validity.

To integrate the second component of the proteomic data, we identify significant protein quantity changes based on the p-value of the ANOVA test used in the initial analysis of the proteomic data as implemented in OpenSWATH^15^. As we encounter a multiple-testing problem when evaluating multi-protein complexes, we apply the Bonferroni correction to adjust the p-value threshold. Any protein quantity change that passes this threshold is regarded as significant and applied to the flux boundaries of the FVA.

Significant protein quantity changes are applied to both upper and lower flux boundaries to ensure recursive behavior: identical results should be obtained for sequential increasing and decreasing protein quantity (Fig S4). Our strategy in data integration is based on the primary assumption that the maximal flux through a reaction correlates with the enzyme concentration. However, in some cases a change in protein quantity is not sufficient to describe a change in an organism’s metabolism and regulatory kinetic effects have to be considered. Thus, we regard the protein quantity changes as soft constraints and apply a tolerance level of ± 40% to account for this phenomenon and allow for a smoother integration of the proteomic data. Thus, the new flux bounds are calculated as:


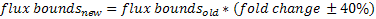


At this point, another flux distribution is computed via FVA representing the model’s solution for the second experimental condition. The difference between the two metabolic states can now be determined based on the comparison of the two flux distributions.

# Supplementary references

1. Fiedler, T. *et al.* Characterization of three lactic acid bacteria and their isogenic ldh deletion mutants shows optimization for YATP (cell mass produced per mole of ATP) at their physiological pHs. *Appl. Environ. Microbiol.* **77,** 612–7 (2011).

2. Mehmeti, I. *et al.* Growth rate-dependent control in Enterococcus faecalis: Effects on the transcriptome and proteome, and strong regulation of lactate dehydrogenase. *Appl. Environ. Microbiol.* **78,** 170–176 (2012).

3. Gillet, L. C. *et al.* Targeted data extraction of the MS/MS spectra generated by data-independent acquisition: a new concept for consistent and accurate proteome analysis. *Mol. Cell. Proteomics* **11,** O111.016717 (2012).

4. Schubert, O. *et al.* Building high-quality assay libraries for targeted analysis of SWATH MS data. *Nat. Protoc.* **10,** 426–441 (2015).

5. Keller, A., Eng, J., Zhang, N., Li, X. & Aebersold, R. A uniform proteomics MS/MS analysis platform utilizing open XML file formats. *Mol. Syst. Biol.* **1,** 2005.0017 (2005).

6. Lam, H. *et al.* Development and validation of a spectral library searching method for peptide identification from MS/MS. *Proteomics* **7,** 655–667 (2007).

7. Craig, R. & Beavis, R. C. A method for reducing the time required to match protein sequences with tandem mass spectra. *Rapid Commun. Mass Spectrom.* **17,** 2310–2316 (2003).

8. MacLean, B., Eng, J. K., Beavis, R. C. & McIntosh, M. General framework for developing and evaluating database scoring algorithms using the TANDEM search engine. *Bioinformatics* **22,** 2830–2832 (2006).

9. Eng, J. K., Jahan, T. a. & Hoopmann, M. R. Comet: An open-source MS/MS sequence database search tool. *Proteomics* **13,** 22–24 (2013).

10. Keller, A., Nesvizhskii, A. I., Kolker, E. & Aebersold, R. Empirical statistical model to estimate the accuracy of peptide identifications made by MS/MS and database search. *Anal. Chem.* **74,** 5383–5392 (2002).

11. Shteynberg, D. *et al.* iProphet: Multi-level Integrative Analysis of Shotgun Proteomic Data Improves Peptide and Protein Identification Rates and Error Estimates. *Mol. Cell. Proteomics* **10,** M111.007690–M111.007690 (2011).

12. Reiter, L. *et al.* Protein identification false discovery rates for very large proteomics data sets generated by tandem mass spectrometry. *Mol. Cell. Proteomics* **8,** 2405–2417 (2009).

13. Escher, C. *et al.* Using iRT, a normalized retention time for more targeted measurement of peptides. *Proteomics* **12,** 1111–1121 (2012).

14. Lam, H. *et al.* Building consensus spectral libraries for peptide identification in proteomics. *Nat. Methods* **5,** 873–875 (2008).

15. Röst, H. L. *et al.* OpenSWATH enables automated, targeted analysis of data-independent acquisition MS data. *Nat. Biotechnol.* **32,** 219–23 (2014).

16. Reiter, L. *et al.* mProphet: automated data processing and statistical validation for large-scale SRM experiments. *Nat. Methods* **8,** 430–435 (2011).

17. Choi, M. *et al.* MSstats: an R package for statistical analysis of quantitative mass spectrometry-based proteomic experiments. *Bioinformatics* **30,** 1–2 (2014).

18. Veith, N. *et al.* Using a Genome-Scale Metabolic Model of Enterococcus faecalis V583 To Assess Amino Acid Uptake and Its Impact on Central Metabolism. *Appl. Environ. Microbiol.* **81,** 1622–33 (2015).

19. Olivier, B. G., Rohwer, J. M. & Hofmeyr, J.-H. S. Modelling cellular systems with PySCeS. *Bioinformatics* **21,** 560–1 (2005).
